# An Epitope-Substituted DNA Vaccine Improves Safety and Immunogenicity against Dengue Virus Type 2

**DOI:** 10.1371/journal.pntd.0003903

**Published:** 2015-07-02

**Authors:** Chung-Tao Tang, Pi-Chun Li, I-Ju Liu, Mei-Ying Liao, Chiung-Yi Chiu, Day-Yu Chao, Han-Chung Wu

**Affiliations:** 1 Graduate Institute of Life Sciences, National Defense Medical Center, Taipei, Taiwan; 2 Institute of Cellular and Organismic Biology, Academia Sinica, Taipei, Taiwan; 3 Graduate Institute of Microbiology and Public Health, College of Veterinary Medicine, National Chung Hsing University, Taichung, Taiwan; Florida Gulf Coast University, UNITED STATES

## Abstract

Dengue virus (DENV), a global disease, is divided into four serotypes (DENV1-4). Cross-reactive and non-neutralizing antibodies against envelope (E) protein of DENV bind to the Fcγ receptors (FcγR) of cells, and thereby exacerbate viral infection by heterologous serotypes via antibody-dependent enhancement (ADE). Identification and modification of enhancing epitopes may mitigate enhancement of DENV infection. In this study, we characterized the cross-reactive DB21-6 and DB39-2 monoclonal antibodies (mAbs) against domain I-II of DENV; these antibodies poorly neutralized and potently enhanced DENV infection both *in vitro* and *in vivo*. In addition, two enhancing mAbs, DB21-6 and DB39-2, were observed to compete with sera antibodies from patients infected with dengue. The epitopes of these enhancing mAbs were identified using phage display, structural prediction, and mapping of virus-like particle (VLP) mutants. N8, R9, V12, and E13 are the reactive residues of DB21-6, while N8, R9, and E13 are the reactive residues of DB39-2. N8 substitution tends to maintain VLP secretion, and decreases the binding activity of DB21-6 and DB39-2. The immunized sera from N8 substitution (N8R) DNA vaccine exerted greater neutralizing and protective activity than wild-type (WT)-immunized sera, both *in vitro* and *in vivo*. Furthermore, treatment with N8R-immunized sera reduced the enhancement of mortality in AG129 mice. These results support identification and substitution of enhancing epitope as a novel strategy for developing safe dengue vaccines.

## Author Summary

Dengue virus (DENV) infects 390 million humans annually, and is the cause of one of the most important arthropod-borne viral diseases in the world. Currently, there are no available licensed vaccines or antiviral drugs for dengue, so development of safe vaccine and effective therapy is urgently needed. Here, we identified two monoclonal antibodies, DB21-6 and DB39-2, which can enhance DENV1-4 infection and increase virus-induced mortality in AG129 mice. We found that serum samples from patients with severe dengue disease contain higher levels of antibodies against enhancing epitope. We proceeeded to identify enhancing epitope on E protein, and developed DNA vaccines by substitution. The substituted DNA vaccine with mutation at the enhancing epitope demonstrated augmented neutralizing activity against DENV2, and reduced enhancement of mortality as compared to wild type-immunized sera. Our results show that substitution of enhancing epitope can increase the immune response against viral infection, while reducing the potential for antibody-dependent enhancement (ADE). These novel findings may be useful for developing safe and efficacious vaccines against dengue.

## Introduction

Dengue virus (DENV) is a mosquito-borne virus that causes prevalent, global disease. It is estimated to cause 390 million infections annually, which lead to a spectrum of clinical syndromes ranging from dengue fever (DF) to severe dengue hemorrhagic fever (DHF) and dengue shock syndrome (DSS) in tropical and subtropical countries [1,2]. Although primary infection with DENV provides immunity against the same serotype, subsequent secondary infection with different DENV serotypes has a higher risk for developing severe dengue disease [3–5]. The presence of cross-reactive and non-neutralizing antibodies bound to DENV helps viral entry into Fcγ receptor (FcγR)-bearing cells, resulting in increased virus load and/or production of certain cytokines [6]. This phenomenon is termed antibody-dependent enhancement (ADE) [3,7]. At the time of writing, there is no approved vaccine against DENV infection [8].

DENV, which exists as four related serotypes (DENV1-4), is a member of the *Flavivirus* genus within the *Flaviviridae* family [9]. It is a positive-stranded RNA virus that encodes three structural proteins (the capsid (C), pre-membrane (prM), and envelope (E) proteins) and seven non-structural proteins (NS1, NS2a, NS2b, NS3, NS4a, NS4b and NS5) [10,11]. The prM protein seems to function as a chaperone for the assembly of the E protein [12]. During virus maturation, the viral particle is activated by the low pH of the trans-Golgi network (TGN). The prM protein is subsequently cleaved by furin to generate M protein, resulting in mature and infective virions [13]. Co-expression of prM and E proteins can produce virus-like particles (VLPs), which are similar in structure and antigenicity to infectious virus particles, and have been used broadly in epitope mapping, diagnosis, and vaccine development [14–16].

The E protein plays an important role in facilitating attachment of DENV to cell surface receptor(s), fusion of virus with endosomal membranes, and subsequent entry into target cells. The E protein is regarded to be the antigen involved in mediating the immune response, and the principle target of neutralizing antibodies [17]. The E protein forms 90 homodimers on the surface of the mature virion [18]. The E monomer consists of three domains: domain I (EDI), domain II (EDII), and domain III (EDIII) [19]; EDI links EDII with EDIII. EDII is an elongated dimerization domain, which contains the conserved fusion peptide [20]. EDIII, an immunoglobulin (Ig)-like domain, is considered to be the binding site of the receptor on the target cell. Several studies have shown that serotype-specific and neutralizing mouse monoclonal antibodies bind to EDIII [21–23], whereas in human, only a small fraction of antibodies react to this region [24,25]. Recent study has shown that human neutralizing antibodies bind to complex epitopes on dengue virions [24]. However, a large fraction of cross-reactive and weakly neutralizing human antibodies can be isolated from natural DENV infection [26,27]. In the context of dengue pathogenesis, these cross-reactive and non-neutralizing antibodies against E or prM proteins derived from primary infection can enhance viral infection through ADE during secondary infection [26]. Therefore, identification of B-cell epitopes of DENV E protein, which induce cross-reactive and non-neutralizing antibodies, may provide valuable information for vaccine development.

Although various strategies have been employed in an attempt to develop dengue vaccine (including the use of attenuated or inactive virus, and the development of subunit vaccines), a safe and effective vaccine against DENV is not yet available [28]. Thus, there is a need to identify and substitute the epitopes recognized by poorly neutralizing and highly enhancing antibodies to improve the dengue vaccine. In this study, we found that the cross-reactive mAbs DB21-6 and DB39-2 exhibit poor neutralizing activity and high capacity for enhancing DENV infection. We used competitive enzyme-linked immunosorbent assay (ELISA) to determine the relationship between mAbs and sera antibodies from dengue patients. We proceeded to use phage display, bioinformatic analysis, and VLP mutants to identify the epitopes recognized by DB21-6 and DB39-2. To further improve the DNA vaccines against DENV2, we substituted the N8 residue of wild-type (WT) DENV2 E protein with arginine (N8R) in a plasmid for immunization. N8R-immunized sera produced higher neutralizing and protective activity than WT-immunized sera. Moreover, treatment of AG129 mice with N8R-immunized sera reduced mortality, as compared with mice treated with WT-immunized sera. Taken together, we have identified a novel cross-reactive and infection-enhancing epitope in E protein. Our results demonstrate that substitution of this enhancing epitope is a promising strategy for development of a safe dengue vaccine.

## Materials and Methods

### Ethics statement

Mouse experiments were carried out in accordance with strict guidelines from the Care and Use Manual of the National Laboratory Animal Center, Taiwan. The protocol was approved by the Committee on the Ethics of Animal Experiments of Academia Sinica (Permit Number: 11-04-166). The human serum samples were collected during an outbreak between 2002 and 2003 in Taiwan. The study protocol was approved by the National Taiwan University Institutional Review Board (NTUH-REC No. 200903086R). The written informed consent was obtained, and all human serum samples were coded for anonymity.

### DENV, cell lines, and mAbs

Four dengue virus serotypes, DENV1 Hawaii, DENV2 16681, DENV3 H87, and DENV4 H241, were prepared as previously described [29]. C6/36 cells were grown in medium consisting of 50% Mitsumashi and Maramorsch insect medium (Sigma-Aldrich) plus 50% Dulbecco’s modified Eagle’s medium (DMEM, Gibco) containing 10% fetal bovine serum (FBS, Gibco) and 100 U/ml penicillin, 100 μg/ml streptomycin, and 0.25 μg/ml amphotericin B (Antibiotic-Antimycotic, Gibco). The C6/36 cells were infected with DENV at a multiplicity of infection (MOI) of 0.1–1, and incubated at 28°C for 7 to 9 days. The viruses were harvested from supernatant, and then titrated in a baby hamster kidney fibroblast cell line (BHK-21) by plaque assay. The aliquots were stored at -80°C. BHK-21 cells were grown in minimal essential medium (MEM, Gibco) supplemented with 10% FBS, 100 U/ml penicillin, 100 μg/ml streptomycin, and 0.25 μg/ml amphotericin B (Antibiotic-Antimycotic, Gibco). Human erythroleukaemic K562 and monocytic THP-1 cells were grown in RPMI medium (Gibco) containing 10% FBS. The mouse mAbs, including DB21-6 and DB39-2, were generated by immunization of BALB/c mice with DENV2, and were produced in hybridoma cells, as previously described [21]. DB21-6 and DB39-2 were isotyped as IgG1 (SouthernBiotech) and purified using protein G Sepharose 4B gels (GE Healthcare).

### *In vitro* measurement of ADE with mAbs

Serial dilutions of mAbs were incubated with DENV1 Hawaii (MOI = 1), DENV2 16681 (MOI = 1), DENV3 H87 (MOI = 5), and DENV4 H241 (MOI = 1) for 1 hour at 4°C. The mixtures were then used to infect K562 cells for 2 hours at 37°C. After washing, the cells were incubated with 2% FBS in RPMI medium (Gibco) at 37°C for 3 days. The infected cells were collected and fixed with 3.7% formaldehyde for 10 minutes at 4°C. For staining, the cells were permeabilized with 2% FBS in PBS containing 0.1% saponin (Sigma), followed by staining with 4 μg/ml 4G2 for 0.5 hours at 4°C. The cells were washed and incubated with R-phycoerythrin (RPE)-conjugated goat anti-mouse IgG (Jackson ImmunoResearch Laboratories) for 1 hour at 4°C. The cells were washed, and the percentages of infected cells were determined by flow cytometry. For infection of THP-1 cells, DENV2 16681 (MOI = 1 or 10) was incubated with diluted mAbs for 1 hour at 4°C, and then incubated with cells for 2 hours at 37°C. After 3 days, the cells were fixed, permeabilized, and stained with hDB32-6 [21]. After washing, the cells were incubated with an RPE-conjugated goat anti-human IgG (Jackson ImmunoResearch Laboratories), and were subsequently analyzed by flow cytometry.

### Measurement of *in vivo* ADE with mAbs in AG129 mice

Type I and II interferon receptor-deficient mice (AG129; 5- to 6-weeks-old) were purchased from B&K Universal. The AG129 mice were given intraperitoneal (i.p.) injections of 5 μg mAbs in 200 μl PBS on days -1 and 1. The mouse IgG1 isotype antibody was used as a negative control. On day 0 of infection, mice were intravenously (i.v.) inoculated with 1 × 10^5^ pfu of the mouse-adapted DENV2 S221 (obtained from Sujan Shresta) [30], in 100 μl PBS. The survival rates of AG129 mice were recorded for 30 days.

### Measurement of viremia by quantitative RT-PCR

AG129 mice were infected with 1 × 10^5^ pfu DENV2 S221 by i.v. inoculation on day 0, and treated with 5 μg mAbs via i.p. injection on days -1 and 1. Viral RNA was extracted from pooled and infected mice sera using the QIAamp viral RNA minikit (Qiagen). Quantitative RT-PCR was performed based on previously published procedures [31], using the LightCycler 480 system (Roche). The standard curve was generated with DENV2 S221 (at concentrations from 10^1^ to 10^7^ pfu/ml). Viremia measurements were expressed as pfu equivalents/ml, which was calculated based on the threshold cycle value (Ct) according to the standard curve for DENV2 S221.

### Competitive ELISA of mAbs and patient serum samples

A total of 21 DENV2-infected patient serum samples were collected from 11 DF patients and 10 DHF patients during an outbreak between 2002 and 2003 in Taiwan. Diagnosis of DENV infection was based on IgM antibody-capture ELISA (MAC-ELISA), reverse-transcriptase PCR (RT-PCR), or virus isolation in cell cultures, as previously described [15]. These serum samples were collected between days 4 and 22 from the onset of symptoms; such sera contained anti-dengue antibodies. All of these patients were determined to have classical DF or DHF based on the criteria published by the World Health Organization (WHO) in 2009 [32]. The characteristics of patient serum samples enrolled in this study are also provided (S1 Table). Competitive ELISA was performed as previously described [33]. Briefly, the plates were coated with polyclonal rabbit anti-DENV hyper-immune sera at 4°C overnight. After blocking, the diluted DENV2 viral supernatants (1 × 10^6^ pfu) were added for 2 hours at room temperature (RT). The diluted mAbs and patient sera (1:100 dilution) were incubated for 2 hours at RT. After washing, horseradish peroxidase (HRP)-conjugated anti-mouse IgG (Jackson ImmunoResearch Laboratories) was added for 1 hour at RT. The peroxidase substrate *o*-phenylenediamine dihydrochloride (OPD, Sigma-Aldrich) was then added, and the reaction was stopped with 3N HCl. The optical density (OD) was measured at 490 nm. Normal human serum (NHS) was used as a control. The percentage of competition was calculated as follows: competition (%) = [1−(OD of patient serum-mAb mixture/OD of NHS-mAb mixture)] × 100.

### Phage display biopanning

Phage display biopanning was performed as previously described [21]. Briefly, the plate was coated with 100 μg/ml mAbs at 4°C for 6 hours. After washing and blocking, 4 × 10^10^ pfu of phage-displayed peptide library (New England BioLabs, Inc.) were incubated for 50 mins at RT. After washing, bound phage was eluted with 100 μl 0.2 M glycine/HCl (pH 2.2) and neutralized with 15 μl 1 M Tris/HCl (pH 9.1). The eluted phage was then amplified in ER2738 for subsequent rounds of selection. The phage was titrated onto LB plates containing IPTG and X-Gal. The second and third rounds of selection were identical to the first round except for the addition of 2 × 10^11^ pfu of amplified phage.

### Identification of immunopositive phage clones by ELISA

The plate was coated with 50 μg/ml mAbs. After washing and blocking, the amplified phages were added, and incubated for 1 hour at RT. After washing, diluted HRP-conjugated anti-M13 antibody (GE Healthcare) was added at RT for 1 hour. The plates were developed, and subsequently terminated by 3N HCl. The OD was measured at 490 nm.

### Identification of epitopes using flow cytometry-based binding assay to cells expressing WT and mutant DENV2 prM/E proteins

The pCBD2-2J-2-9-1 plasmid expressing prM-E proteins of DENV2 has been previously characterized and described [14–16]. Site-directed mutagenesis was performed to replace each of the selected amino acid residues, as described in the previous study [21]. After mutagenesis, the plasmids were sequenced to ensure the absence of any further mutations at non-target sites. BHK-21 cells were transfected with constructs expressing the wild-type (WT) or mutant DENV2 E protein using polyjet *in vitro* DNA transfection reagent (SignGen Laboratories). After 2 days, the cells were fixed, and permeabilized with 2% FBS in PBS containing 0.1% saponin (Sigma). For staining, cells were incubated with DB21-6, DB39-2, 4G2, and mixed mAbs (DB32-6, 3H5, and DB25-2) at a concentration of 1, 1, 1, and 1 μg/ml, respectively, at 4°C for 0.5 hours. After washing, the cells were incubated with RPE-conjugated goat anti-mouse IgG (Jackson ImmunoResearch Laboratories), and analyzed by flow cytometry. The relative index of a mAb to a mutant E protein was measured using the formula: [intensity of the mutant E/intensity of WT E (recognized by a mAb)]/[intensity of mutant E/intensity of WT E (recognized by mixed mAbs)].

### Detection of secreted VLPs by capture ELISA

BHK-21 cells were transfected with vectors expressing the WT or mutant E protein of DENV2, as described above. At 48 hours post-transfection, culture supernatants were collected. The plates were coated with polyclonal rabbit anti-DENV hyper-immune sera at 4°C overnight. After blocking, two-fold dilutions of supernatants containing WT or mutant VLPs were added for 2 hour at RT. The wells were then incubated with diluted DB32-6 and 4G2 at RT for 2 hour. After washing, a 1:2000 dilution of HRP-conjugated anti-mouse IgG (Jackson ImmunoResearch Laboratories) was added for 1 hour at RT. Finally, the plates were developed, and the reaction was subsequently terminated with 3N HCl. The OD was measured at 490 nm.

### Preparation of plasmids for immunization

Plasmids expressing WT E protein of DENV2 or a mutant E protein in which the N8 residue was substituted with R (N8R) were used for immunization. For coating, 25 mg of 1.0 μm gold powder was resuspended with 50 mM spermidine (Sigma-Aldrich, St. Louis, MO). Then, 50 μg of plasmid DNA was added, followed by the addition of 1M CaCl_2_ (Sigma-Aldrich, St. Louis, MO); the solution was mixed and precipitated for 10 mins at RT. After collection by centrifugation, the gold-DNA complex was washed with absolute ethanol and resuspended in 0.1 mg/ml of polyvinylpyrrolidone (PVP) (360 kDa; Sigma Chemicals, Inc.) solution. The slurry was injected into a TefzelR tube (McMaster-Carr, Chicago, IL), and then coated. After the ethanol had dried off, the tube was cut into 0.5-inch bullets and stored at -20°C. The gold in each bullet contained 1 μg of DNA. Before use, the bullets were loaded into the Helios gene gun device (Bio-Rad, Hercules, CA) for delivery of plasmids.

### Immunization of mice

The abdominal epidermis of 6 week-old female BALB/c mice was injected with a gene gun using a helium pressure setting of 400 lb/inch^2^. Each mouse was immunized by administering 4 bullets containing 1 μg plasmid DNA. Mice were immunized at 0, 3, and 6 weeks. Serum samples were collected before immunization and 3 weeks after the third immunization (pre-, 1^st^, 2^nd^, 3^rd^ immunized sera). The serum samples were pooled from five to six mice for each immunized group and evaluated by ELISA, neutralization assay, and *in vivo* ADE assay.

### Evaluation of immunized sera against DENV2 by ELISA

C6/36 cells infected with DENV2 16681 were used as antigens. C6/36 cells were seeded into each well (2 × 10^4^ cells/well) of 96-well ELISA plates. After one day, 2 × 10^3^ pfu of DENV2 16681 (MOI = 0.1) was added to infect the cells at 37°C for 2 hours. The wells were washed with PBS, and then cultured in 2% FBS culture medium at 28°C for 5 days. Next, the infected cells were fixed with 1:1 methanol/acetone at 4°C for 10 mins. The plates were blocked with 5% skimmed milk at 4°C for 24 hours. Diluted immunized sera were then added for incubation at RT for 2 hours. The plates were then washed three times with phosphate-buffered saline containing 0.1% (w/v) Tween 20 (PBST_0.1_), and subsequently incubated with HRP-conjugated anti-mouse IgG (Jackson ImmunoResearch Laboratories). Finally, the plates were developed, and the reaction terminated with 3N HCl. The OD was measured at 490 nm.

### *In vitro* and *in vivo* neutralization assays with immunized sera

DENV2 16681 (MOI = 1) was incubated with 3^rd^ immunized sera for 1 hour at 4°C. Next, the mixtures were used to infect BHK-21 cells for 2 hours at 37°C. After 3 days, the cells were fixed, permeabilized, and stained with 4 μg/ml 4G2. After washing, the cells were incubated with RPE-conjugated goat anti-mouse IgG (Jackson ImmunoResearch Laboratories), and analyzed by flow cytometry. Inhibition percentage (%) = [1−(the percentage of infected cells incubated with immunized sera/without immunized sera)] × 100.

The ICR mice were purchased from the Laboratory Animal Center, National Taiwan University College of Medicine. Serially-diluted immunized sera were incubated with 1 × 10^4^ pfu (25-fold lethal dose, 25-fold LD_50_) of DENV2 16681 for 0.5 hours at 4°C. Two-day-old suckling mice were inoculated with 20 μl of the mixtures through intracranial (i.c.) injection. After challenge, the survival rates were recorded for 28 days.

### Studies of *in vivo* ADE with immunized sera

AG129 mice were given i.p. injections of dilutions of immunized sera on days -1 and 1, and were i.v. inoculated with 1 × 10^5^ pfu of DENV2 S221 on day 0. The survival rates were recorded for 30 days.

### Statistical analysis

Survival rate was expressed using Kaplan-Meier survival curves, and statistical analyses were performed using GraphPad Prism 5. For competition assays of mAbs and patient sera, Student’s *t* tests were used to identify significant differences and calculate *P* values (*P<0.05, ***P<0.001, NS not significant). For evaluation of immunized sera against DENV2 by ELISA, two-way ANOVA with Bonferroni *post-hoc* test was used to determine the significant differences and calculate *P* values (**P<0.01, NS not significant). GraphPad Prism 5 was used to analyze 50% inhibition titers against DENV2, based on inhibition percentages from pooled immunized sera.

## Results

### Characterization of cross-reactive DB21-6 and DB39-2 against DENV

In our previous study, we generated seventeen mAbs against the E protein of DENV [21]. Of these mAbs, DB21-6 and DB39-2 could recognize cells infected with DENV1-4 (S1A Fig). In addition, these mAbs recognized transfected BHK-21 cells expressing DENV2 E and EDI-II proteins (S1B and S1C Fig). Thus, the cross-reactive DB21-6 and DB39-2 recognized DENV1-4 and domain I-II on E protein.

To estimate the *in vitro* neutralizing activity, we infected BHK-21 cells with a mixture of individual mAbs and DENV1-4. Previous studies have reported that 4G2 is an anti-flavivirus antibody with neutralizing and enhancing activity at certain concentrations [16]. We observed that 4G2 exerts higher neutralization activity than DB21-6 and DB39-2 against DENV2 (S2 Fig). In addition, DB21-6 and DB39-2 exhibited non-neutralizing activity against DENV1-4 (50% inhibition concentration, >33 μg/ml) (S2B Fig).

### Enhancing activities of DB21-6 and DB39-2

**Fig 1:**
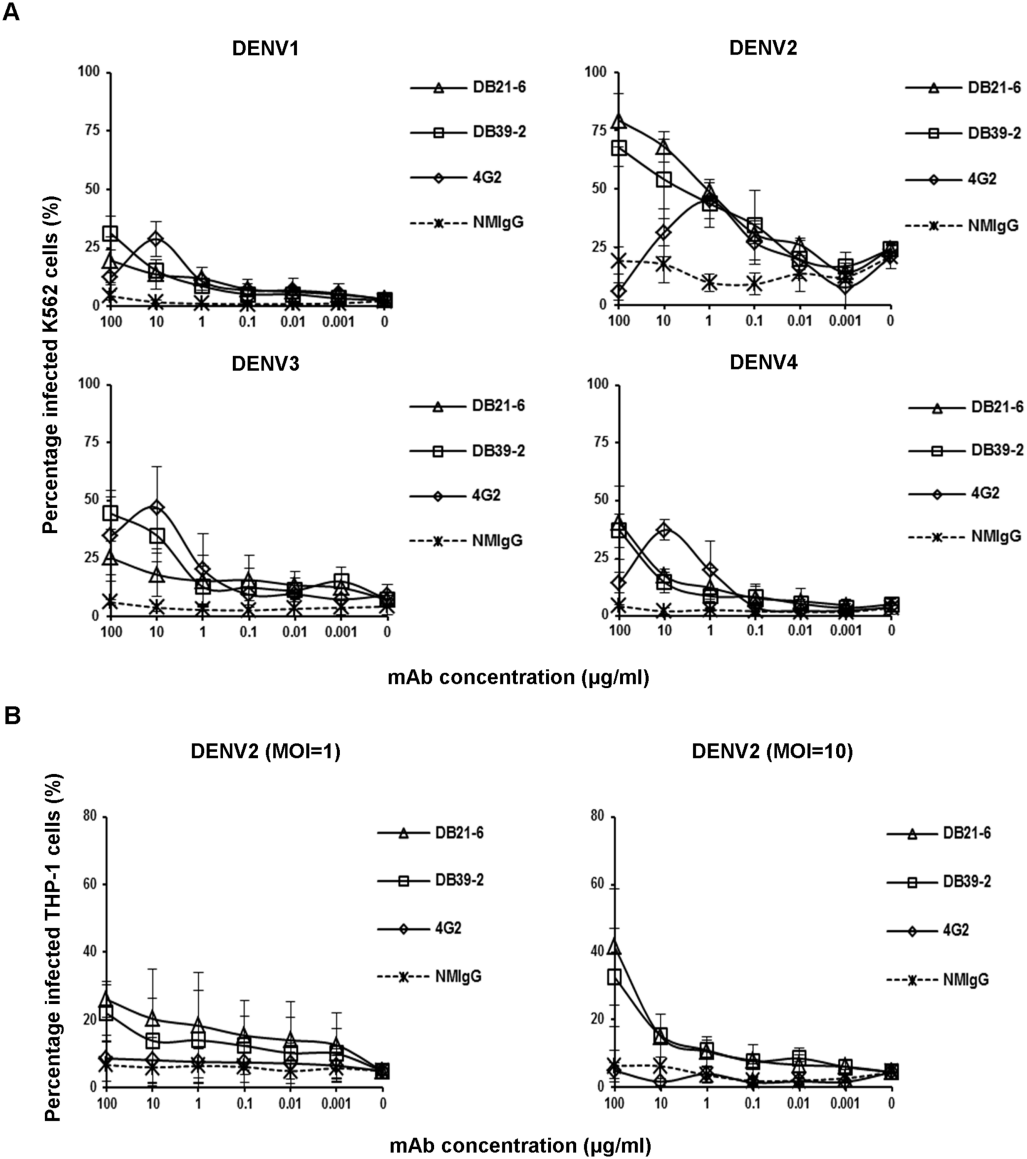
ADE of DENV mediated by cross-reactive mAbs. (A) Infection of K562 cells with DENV1 (MOI = 1), DENV2 (MOI = 1), DENV3 (MOI = 5), or DENV4 (MOI = 1) in the presence of serially-diluted mAbs was examined at 72 hours post-infection by staining with 4G2 and RPE-conjugated goat anti-mouse IgG, followed by flow cytometry. The percentages of infected K562 cells are shown. Data shown are the mean ± standard error of the mean (SEM) from three independent experiments. (B) The indicated mAbs were serially diluted and incubated with DENV2 (MOI = 1 or 10) at 4°C for 1 hour. The mixtures were then added to THP-1 cells, and the infected cells were examined by flow cytometry at 72 hours post-infection. The infected cells were stained with hDB32-6 and RPE-conjugated goat anti-human IgG. The percentage of infected THP-1 cells is shown. NMIgG was used as a control. Data shown are the mean ± SEM from three independent experiments.

To investigate *in vitro* enhancement of DENV infection through ADE [34,35], we performed *in vitro* ADE assays, and detected the increases in the percentage of dengue-infected cells by flow cytometry [36]. The FcγRIIA-bearing K562 cells, which do not express type 1 interferon (IFN) [37], were used to measure the enhancement of infected cells through extrinsic ADE. The serially-diluted mAbs were incubated with DENV1-4, and then used to infect K562 cells. The infection percentage was measured by flow cytometry, revealing infection enhancement over a broad range of mAb concentrations (Fig 1A). As compared to the other mAbs, 4G2 caused enhancement of DENV1-4 infection in K562 cells at lower antibody concentrations. DB21-6 and DB39-2 enhanced DENV1-4 infection in K562 cells at high antibody concentrations (Fig 1A). To further confirm enhancement of infection, we proceeded to examine the enhancement of DENV2 16681 infection by DB21-6 and DB39-2 in FcγRI- and FcγRIIA-bearing THP-1 cells. Infection in THP-1 cells was enhanced to a greater extent by DB21-6 and DB39-2 than by 4G2 (Fig 1B).

**Fig 2:**
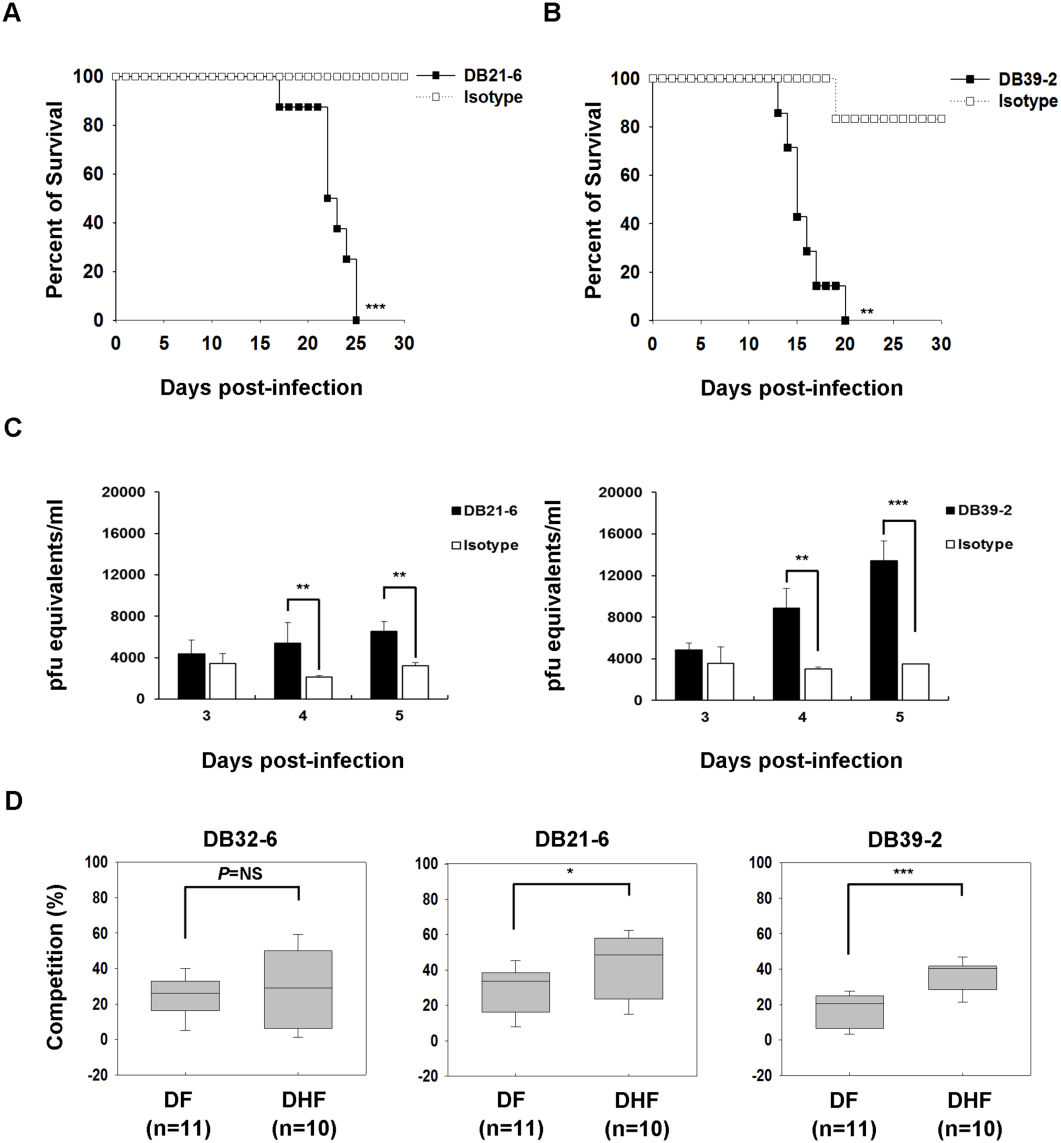
Characterization of *in vivo* ADE and competition of patient sera by DB21-6 and DB39-2 mAbs. (A) AG129 mice were i.v. infected on day 0 with 1 × 10^5^ pfu DENV2 S221, and given i.p. injections of 5 μg of DB21-6 (n = 8) or isotype control (n = 5) on days -1 and 1. Survival rates were recorded for 30 days. Kaplan-Meier survival curves and *P* value is shown (***P<0.001, compared to isotype control). Data shown are from one representative experiment of two independent experiments. (B) AG129 mice were i.v. infected on day 0 with 1 × 10^5^ pfu DENV2 S221, and given i.p. injections of 5 μg of DB39-2 (n = 7) or isotype control (n = 6) on days -1 and 1. Survival rates were recorded for 30 days. Kaplan-Meier survival curves and *P* value is shown (**P<0.01, compared to isotype control). Data shown are from one representative experiment of two independent experiments. (C) The viremia was measured in DENV2 S221-infected AG129 mice following treatment with DB21-6 or DB39-2 mAbs using quantitative RT-PCR as described in Materials and Methods. The viral loads were significantly increased at day 4 and 5 post-infection. Data shown are the mean ± SD. The *P* values (**P<0.01, ***P<0.001) were analyzed using two-way ANOVA with Bonferroni *post-hoc* test. (D) Competition between mAbs and patient sera for binding to DENV2. The percentage of competition is shown. Unpaired Student’s *t* tests were used to calculate *P* values (*P<0.05, ***P<0.001, NS not significant).

DENV2 S221 was previously used to study enhancement of mortality via ADE in AG129 mice [30]. To evaluate the *in vitro* enhancement of DENV2 S221 infection by mAbs, we performed ADE assays using K562 cells and THP-1 cells. As for DENV1-4 infection, high concentrations of DB21-6 and DB39-2 enhanced DENV2 S221 infection in K562 cells (S3A Fig). In addition, DB21-6 and DB39-2 enhanced DENV2 S221 infection in THP-1 cells at high concentrations of antibody (S3B Fig). These results suggest that DB21-6 and DB39-2 can enhance DENV2 S221 infection *in vitro*. Next, we confirmed the *in vivo* enhancing activities in AG129 mice. The AG129 mice treated with 5 μg DB21-6 and infected with DENV2 S221 exhibited increased mortality as compared to control infected mice (Fig 2A). In addition, AG129 mice treated with 5 μg of DB39-2 also exhibited elevated mortality (Fig 2B). In order to determine viremia in DENV2 S221-infected AG129 mice following treatment with DB21-6 or DB39-2, the viral RNA levels were measured by quantitative RT-PCR. The results indicate that viral loads were significantly increased after DB21-6 or DB39-2 treatment of infected AG129 mice, as compared to isotype control Ab treatment (Fig 2C). These results indicate that DB21-6 and DB39-2 have non-neutralizing activities, and enhance mortality in AG129 mice.

### Competition assay of mAbs and infected patient sera

We proceeded to perform competition assay to determine whether sera antibodies from dengue patients compete with mAbs for binding to DENV2. The characteristics of patient serum samples enrolled in this study are provided (S1 Table). The sera antibodies from infected patients were observed to compete with DB21-6 and DB39-2. The competition percentages of DB21-6 and DB39-2 were significantly higher in serum samples from DHF patients than those from DF patients (Fig 2D), while the competition percentage of neutralizing DB32-6 [21] was similar for sera from either DF or DHF patients (Fig 2D). We also performed the same experiment with more concentrated serum (1:50 dilution) or diluted serum (1:200 dilution), and obtained similar results (S4 Fig). These results suggest that serum samples from DHF patients contain higher levels of antibodies, which compete for binding with DB21-6 and DB39-2 mAbs.

### Identification of enhancing epitopes of DB21-6 and DB39-2

**Fig 3:**
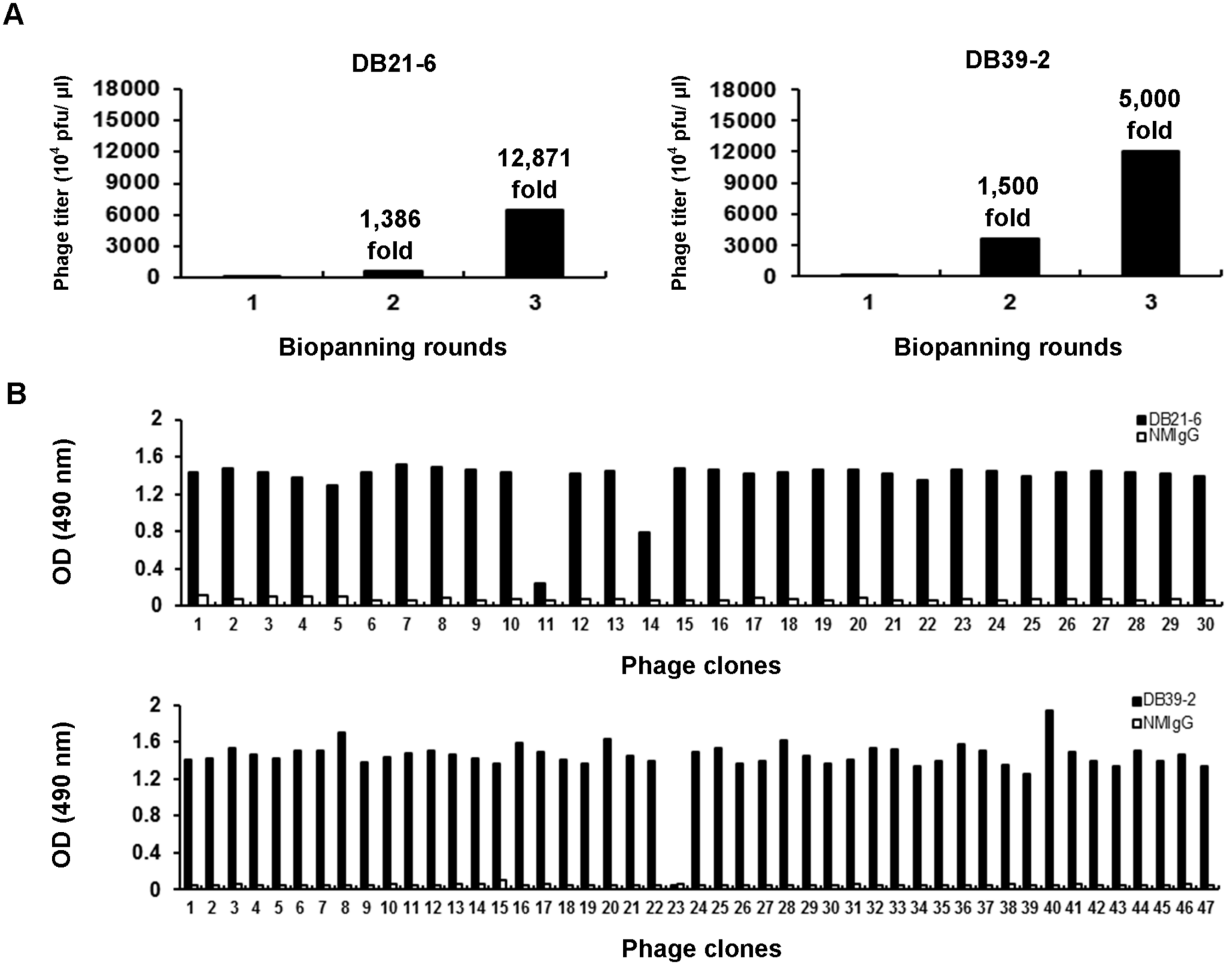
Screening of a phage-displayed peptide library with DB21-6 and DB39-2 mAbs. (A) After 3 rounds of biopanning, phage titers were increased by 12,871-fold (DB21-6) and 5,000-fold (DB39-2), respectively. (B) The immunopositive phage clones selected by DB21-6 and DB39-2 were identified by ELISA. NMIgG was used as a negative control.

In order to identify the enhancing epitopes of DB21-6 and DB39-2, we used a phage-displayed peptide library to screen the reactive phage clones. After three biopanning rounds, the phage titers were increased to 12,871-fold (DB21-6) and 5,000-fold (DB39-2), respectively, compared to that of the first round (Fig 3A). The individual phage clones from the third round of biopanning were randomly selected. As shown by ELISA, most selected phage clones exhibited significant reactivity to the mAbs, but not to normal mouse IgG (NMIgG). Of the 30 selected phage clones, 29 clones reacted with DB21-6 (Fig 3B). The immunopositive phage clones were amplified, and their phage DNA was isolated for DNA sequencing. Eleven phage clones with individual peptide sequences were identified (Table 1). Similarly, of the 47 selected phage clones, 46 reacted with DB39-2 (Fig 3B). Thirteen of the 46 immunopositive phage clones that reacted with DB39-2 possess individual peptide sequences (Table 1). Alignment of peptide sequences revealed the binding motif of DB21-6 and DB39-2 to be N-R-x-x-V-E (Table 1). In addition, modeling of the peptide sequences with the pepitope server (http://pepitope.tau.ac.il/) predicted that the epitope residues on the E protein are N8, R9, V12, and E13 (Table 1).

**Table 1:** Alignment of phage-displayed peptide sequences selected by DB21-6 and DB39-2.

DB21-6 phage clones	Peptide sequences^a^																	
PC21-15				N	G	S	**N**	**R**	D	I	**V**	**E**	V	Q	R			
PC21-10			N	Q	I	Y	**N**	**R**	D	Y	T	**E**	P	T				
PC21-9						Y	**N**	**R**	D	M	L	**E**	T	D	Y	V	N	
PC21-13			Q	N	T	W	**N**	**R**	D	S	I	**E**	E	T				
PC21-17	F	P	E	V	S	V	**N**	**R**	L	V	**V**	**E**						
PC21-20					H	V	**N**	**R**	L	H	**V**	**E**	G	P	V	P		
PC21-18	K	M	T	L	P	M	**N**	**R**	S	H	**V**	**E**						
PC21-2	S	Y	V	T	G	G	**N**	**R**	Y	A	**V**	**E**						
PC21-1		S	S	Y	L	S	**N**	**R**	L	F	T	**E**	A					
PC21-16	S	A	T	T	M	S	**N**	**R**	Y	Y	T	**E**						
PC21-5				Q	P	Y	**N**	**R**	S	Y	I	D	F	M	V			
DENV1-4^b^							**N^8^**	**R**	D	F	**V**	**E^13^**						
DB39-2 phage clones	Peptide sequences^a^																	
DB39-38					L	S	**N**	**R**	L	H	**V**	**E**	S	L	E	L		
DB39-40				N	Q	T	**N**	**R**	H	F	**V**	**E**	I	V	H			
DB39-11		S	G	L	D	R	**N**	**R**	Q	L	**V**	**E**	R					
DB39-39							**N**	**R**	T	L	**V**	**E**	L	G	Y	A	M	L
DB39-3						V	**N**	**R**	P	W	**V**	**E**	T	T	T	Q	G	
DB39-28		I	V	P	Y	S	**N**	**R**	T	V	T	**E**	T					
DB39-31							**N**	**R**	V	S	N	**E**	P	F	W	D	I	A
DB39-34				D	Y	L	**N**	**R**	S	T	N	**E**	P	A	L			
DB39-36		S	M	P	L	S	G	**R**	A	V	**V**	**E**	G					
DB39-47	H	T	S	L	H	S	G	**R**	N	S	**V**	**E**						
DB39-4	S	S	P	G	V	I	S	**R**	F	L	**V**	**E**						
DB39-43							D	**R**	Y	L	**V**	**E**	Y	S	S	G	R	W
DB39-1			M	P	S	G	G	**R**	F	L	**V**	**E**	G	A				
DENV1-4^b^							**N^8^**	**R**	D	F	**V**	**E^13^**						

^a^ The phage-displayed consensus amino acids are indicated by boldface type.

^b^ The amino acid sequences 8 to 13 in E protein of DENV1-4 were retrieved from GenBank (accession number AIU47321, AAB58782, AAA99437, and AAX48017).

**Fig 4:**
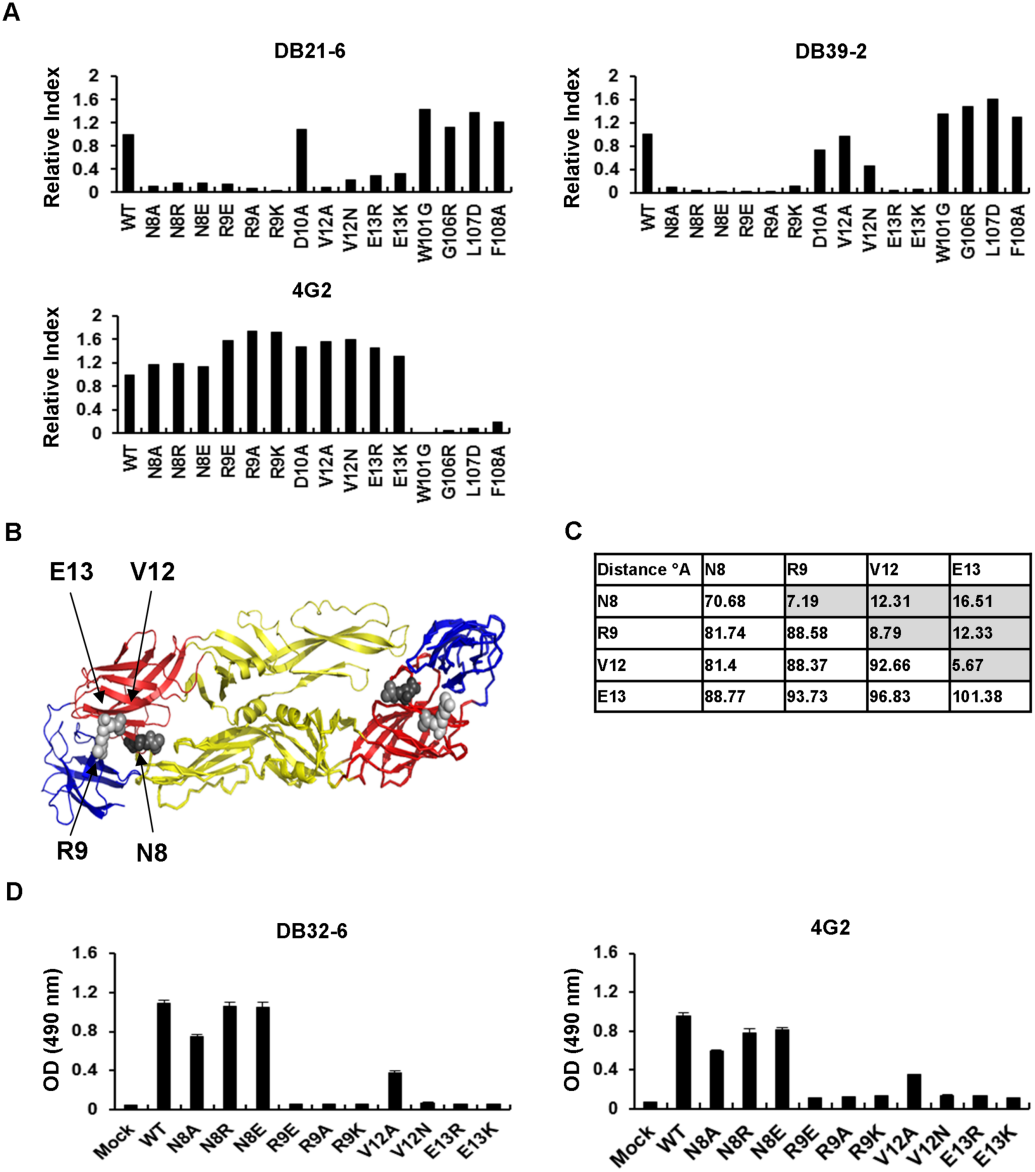
Identification of the epitope residues of DB21-6 and DB39-2 mAbs. (A) BHK-21 cells were transfected with vectors expressing WT or mutant DENV2 E protein. After 2 days, the collected cells were reacted with mAbs, and then analyzed with flow cytometry. The relative indices were measured, as shown. Substitutions of N8, R9, V12, and E13 caused a significant loss of binding activity of DB21-6. Substitutions of N8, R9, and E13 caused a loss of binding activity of DB39-2. 4G2 was used as a control. Data shown are from one representative experiment of two independent experiments. (B) The model is based on the DENV2 E protein model (PDB: 1OAN), with the positions shown from the bottom of the structure. EDI is in red, EDII is in yellow, and EDIII is in blue. The epitopes of cross-reactive mAbs are located at residues N8, R9, V12, and E13 in EDI. (C) Structure analysis determines the distance (°A) between epitope residues from the same (shaded) or adjacent monomer by the PyMOL program. (D) The secreted VLPs were captured by polyclonal rabbit anti-DENV2 E protein sera. The WT and mutant VLPs were detected with DB32-6 and 4G2 by VLP-capture ELISA. Data shown are from one representative experiment of two independent experiments.

To further verify the epitope of DB21-6 and DB39-2, we performed site-directed mutagenesis of the phage-displayed epitope using pCBD2-2J-2-9-1 as template. After confirmation of variants by sequencing, we transfected cells with the mutant plasmids, and detected binding activity by flow cytometry. The binding percentages for each transfectant were normalized to those of anti-EDIII mAbs (DB32-6, 3H5, and DB25-2) [21], and relative indices were calculated (Fig 4A). 4G2, which binds to residues at the fusion loop of EDII [16], was used as a control to verify the structural change of E proteins caused by mutations (Fig 4A). Based on the relative indices, we found that mutations at N8, R9, V12, and E13 prevented binding by DB21-6. The same method was used to identify the epitope residues of DB39-2 as N8, R9, and E13. Structural modeling was applied to show that the recognition residues are located in domain I of E protein (Fig 4B). The distance between these residues from the same monomers was analyzed using a structure modeling program, and was found to be less than 30°A (Fig 4C); interestingly, this distance can be spanned by a single IgG molecule [16]. This suggests that the N8, R9, V12, and E13 residues constitute the epitope of DB21-6. In addition, the N8, R9, and E13 residues constitute the epitope of DB39-2. Alignments revealed that the binding motif of DB21-6 and DB39-2 corresponds to the N8, R9, V12, and E13 residues, which are conserved in DENV1-4 (S2 and S3 Tables). Finally, we used VLP-capture ELISA to demonstrate that the mutations at R9, V12, and E13 affect DENV2 VLP secretion (Fig 4D). The effects of these mutations on the ability to secrete VLPs might be due to a change in the structure of E protein. However, the N8R substitution did not affect DENV2 VLP secretion (Fig 4D). N8 substitution tends to maintain VLP secretion and reduces the binding activity of DB21-6 and DB39-2.

### Examination of humoral immune responses in mice

**Fig 5:**
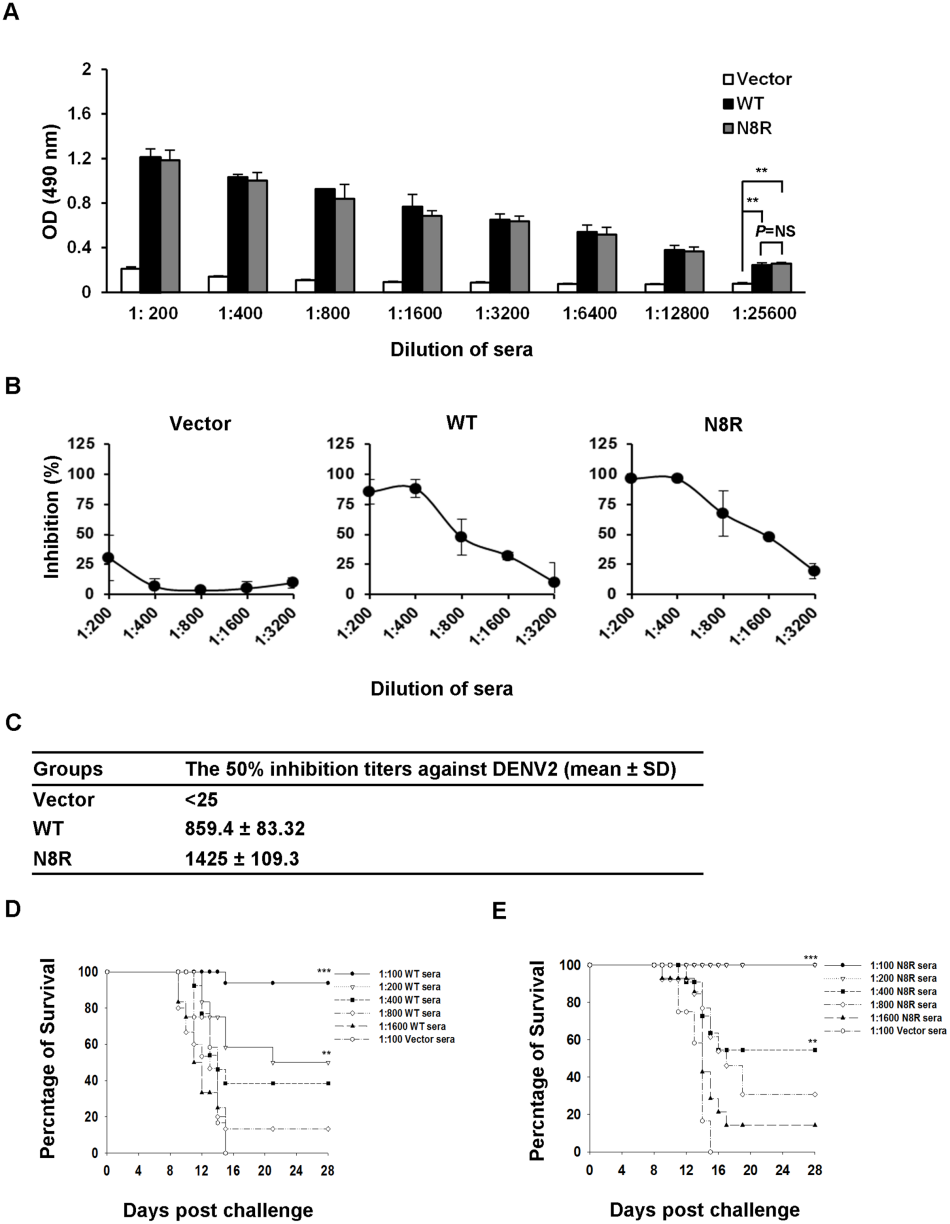
*In vitro* and *in vivo* neutralization assays of DENV2 with immunized sera. (A) Mice were immunized with vector, WT, or N8R plasmids at three-week intervals. After the 3^rd^ immunization, serum samples were collected and pooled for each immunized group. The immunized sera were evaluated by ELISA using plates containing C6/36 cells infected with DENV2 16681. The OD was measured at 490 nm. Data shown are from one representative experiment of two independent experiments. The *P* values (**P<0.01, NS not significant) were analyzed using two-way ANOVA with Bonferroni *post-hoc* test. (B) After the 3^rd^ immunization, the pooled sera were serially diluted and incubated with DENV2 (MOI = 1) for 1 hour at 4°C. The mixtures were then used to infect BHK-21 cells. After 3 days, the cells were stained with 4G2, and analyzed by flow cytometry. Inhibition percentages are shown for the mean of an experiment conducted in triplicate. Data shown are from one representative experiment of two independent experiments. (C) Table showing titers conferring 50% inhibition against DENV2 infection. (D and E) After the 3^rd^ immunization, the pooled sera were serially diluted and incubated with 25-fold LD_50_ of DENV2 for 0.5 hour at 4°C. Next, the mixtures were injected into ICR suckling mice by the intracerebral route (i.c.). The survival rates were recorded for 28 days. The number of animals tested for each immunized sera ranged from 4 to 16 per group. Kaplan-Meier survival curves and *P* values are shown (***P<0.001, **P<0.01, compared to vector-immunized sera). Data shown are from one representative experiment of two independent experiments.

The BALB/c mice were immunized with vector, WT, or N8R plasmids at 0, 3, and 6 weeks. After three rounds of immunization, the serum samples were collected and pooled within each immunized group. Next, the immunized sera were examined by ELISA. A remarkable increase of antibody titer against DENV2 was observed after immunization (S5A Fig). The 3^rd^ WT- and N8R-immunized sera against DENV2 exhibited significantly higher absorbance values than those of vector-immunized sera (Fig 5A). Analysis of immunized sera with anti-IgG1 and IgG2a antibodies revealed that the IgG1/IgG2a ratios increased between the second and third immunization (S5B and S5C Fig). In addition, the immunized mice maintained their anti-DENV2 responses after 15 weeks (S5D Fig).

### Evaluation of neutralizing activity of the immunized sera

The immunized sera were evaluated for their neutralizing activity against DENV2. Both WT- and N8R-immunized sera exhibited high neutralizing activities, while vector-immunized sera did not (Fig 5B). Interestingly, DENV2 infection was more effectively neutralized by N8R-immunized sera than by WT-immunized sera (Fig 5C). To further evaluate whether immunized sera could broadly neutralize the diverse DENV2 strains, BHK-21 cells were infected with mixtures of immunized sera and four different DENV2 strains: 16681, NGC, PL046, and Malaysia 07587. Remarkably, the WT- and N8R-immunized sera exhibited high neutralizing activities against various types of DENV2 strain (S6 Fig).

Next, we examined the protective effect of immunized sera against DENV2 16681 *in vivo*. The survival rates of mice treated with WT-immunized sera at dilutions of 1:100 and 1:200 were significantly higher than that of mice treated with vector-immunized sera at a dilution of 1:100 (Fig 5D), while the survival rates of mice treated with N8R-immunized sera at dilutions of 1:100, 1:200, and 1:400 were significantly higher than that of mice treated with vector-immunized sera at a dilution of 1:100 (Fig 5E). In addition, treatment with WT-immunized sera afforded 50% protection at a dilution of 1:200, while N8R-immunized sera afforded 50% protection at a dilution of 1:400 (Fig 5D and 5E). Hence, N8R-immunized sera possessed higher neutralizing and protective activity than WT-immunized sera both *in vitro* and *in vivo*.

### Reduction of the *in vivo* enhancing activity of the immunized sera

**Fig 6:**
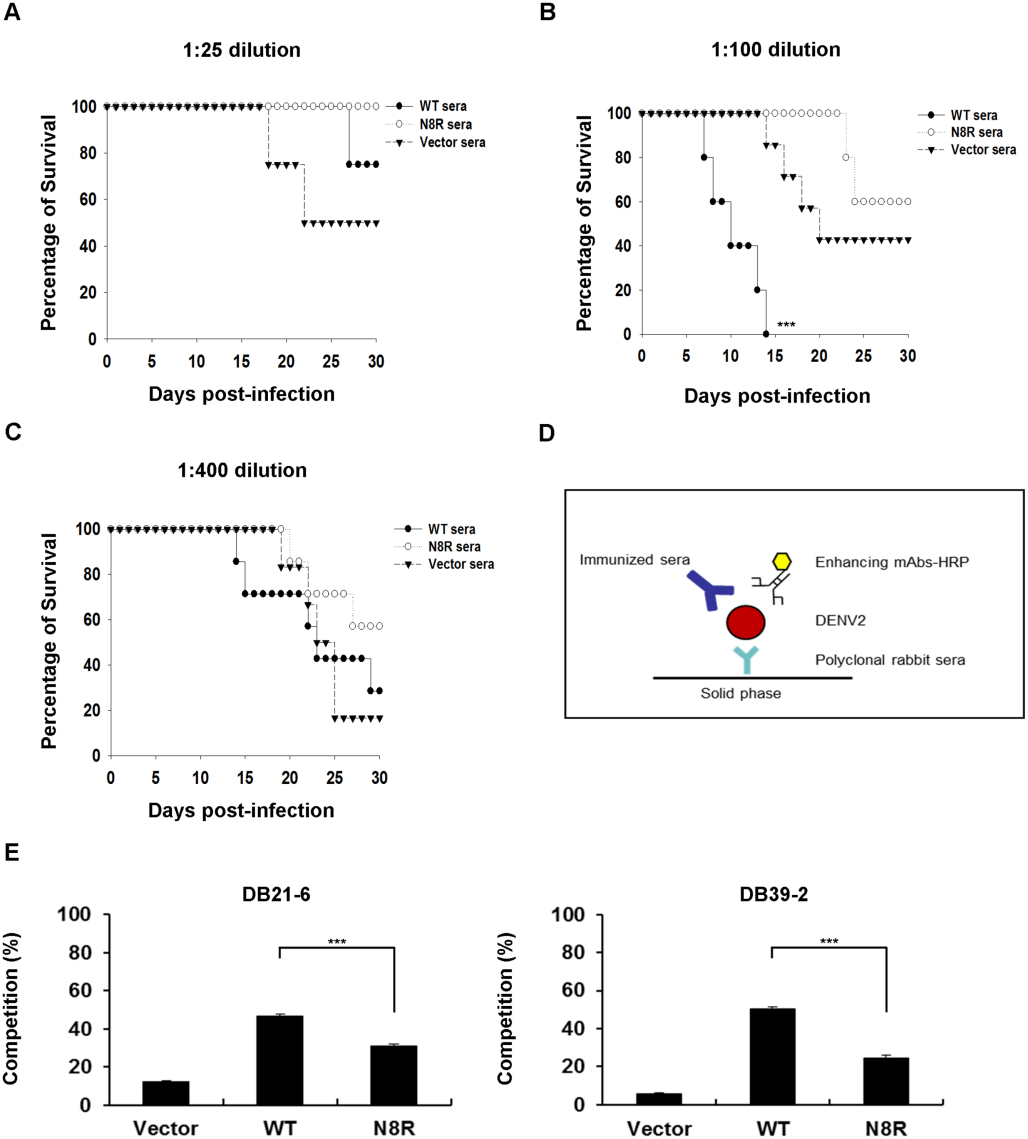
*In vivo* enhancement of mortality in AG129 mice treated with immunized sera. (A) AG129 mice were i.v. infected with 1 × 10^5^ pfu DENV2 S221 on day 0, and given i.p. injections of WT- (n = 4), N8R- (n = 4), or vector- (n = 4) immunized sera (1:25 dilution) on days -1 and 1. Kaplan-Meier survival curves (1:25 dilution) are shown, and *P* values were analyzed using GraphPad Prism 5. The survival rates of mice treated with WT- (*P* = 0.3834) or N8R- (*P* = 0.1278) immunized sera did not significantly differ from that of mice treated with vector-immunized sera. (B) AG129 mice were i.v. infected with 1 × 10^5^ pfu DENV2 (S221) on day 0, and given i.p. injections of WT- (n = 5), N8R- (n = 5), or vector- (n = 7) immunized sera (1:100 dilution) on days -1 and 1. Kaplan-Meier survival curves (1:100 dilution) are shown, and *P* values were analyzed using GraphPad Prism 5. The survival rate of mice treated with WT- (***P = 0.0007) immunized sera was significantly lower than that of mice treated with vector-immunized sera. However, the survival rate of mice treated with N8R- (*P* = 0.3538) immunized sera did not differ from that of mice treated with vector-immunized sera. (C) AG129 mice were i.v. infected with 1 × 10^5^ pfu DENV2 (S221) on day 0, and given i.p. injections with WT- (n = 7), N8R- (n = 7), or vector- (n = 6) immunized sera (1:400 dilution) on days -1 and 1. The survival rates were recorded for 30 days. Kaplan-Meier survival curves (1:400 dilution) are shown, and *P* values were analyzed using GraphPad Prism 5. The survival rate of mice treated with WT- (*P* = 0.8701) or N8R- (*P* = 0.1587) immunized sera did not significantly differ from that of mice treated with vector-immunized sera. (D) Schematic describing the competition assay of mAbs and immunized sera. The ELISA plates were coated with polyclonal rabbit anti-DENV hyper-immune sera at 4°C overnight. After blocking, the diluted DENV2 viral supernatants were added for 2 hours at RT. The HRP-conjugated enhancing mAbs (Innova Biosciences HRP Conjugation Kit) and immunized sera (1:40 dilution) were incubated for 2 hours at RT. After washing, the OPD was added, and the reaction was stopped with 3N HCl. The OD was measured at 490 nm. (E) Competition between mAbs and immunized sera for binding to DENV2. The percentage of competition is shown. Normal mouse serum (NMS) was used as a control. The percentage of competition was calculated as follows: competition (%) = [1−(OD of immunized serum-mAb mixture/OD of NMS-mAb mixture)] × 100. The *P* values (***P<0.001) were analyzed using two-way ANOVA with Bonferroni *post-hoc* test.

In order to study the *in vivo* enhancement of mortality, we passively transferred different dilutions of WT-, N8R-, or vector-immunized sera into AG129 mice. Following infection with DENV2 S221, the survival rate of mice treated with WT- or N8R-immunized sera (1:25 dilution) was higher than that of mice treated with vector-immunized sera (Fig 6A). However, mice treated with WT-immunized sera at a dilution of 1:100 showed higher mortality than mice treated with vector-immunized sera (Fig 6B). Notably, the survival rate of mice treated with N8R-immunized sera at a dilution of 1:100 was higher than that of mice treated with vector-immunized sera (Fig 6B). In addition, no enhancement of mortality was observed in mice treated with N8R-immunized sera (Fig 6B). Finally, treatment with WT- or N8R-immunized sera at a dilution of 1:400 did not have a neutralizing or enhancing effect on the survival rates of mice (Fig 6C). These results indicate that the N8R substitution of E protein can reduce *in vivo* enhancement of mortality.

To further characterize these enhancing antibodies are produced in immunized sera, we performed competitive ELISA to inhibit the binding of HRP-conjugated DB21-6 or DB39-2 mAbs by immunized sera (Fig 6D). The competition percentages of HRP-conjugated DB21-6 and DB39-2 were significantly higher in WT-immunized sera than those in N8R-immunized sera (Fig 6E). These results suggest that N8R substitution would redirect immunodominance by reducing the generation of enhancing antibodies.

## Discussion

DENV infections stimulate immune responses and elicit a small proportion of protective antibodies. However, a high proportion of non-protective antibodies are also generated, which might be associated with enhancement of viral infections. Here, we characterized the ability of DB21-6 and DB39-2 to increase the percentage of dengue virus-infected cells. Furthermore, we confirmed that these mAbs enhance mortality in AG129 mice. Through competition assay, we found that sera antibodies from infected patients compete for binding with these mAbs. Using phage-display, structure prediction, and VLP mutants, we mapped the epitopes of enhancing mAbs DB21-6 and DB39-2 on EDI protein. To investigate how to reduce the enhancing effects while maintaining neutralizing activity, we substituted the N8 residue of E protein, and immunized mice with WT or N8R plasmids with a gene gun delivery system. After three immunizations, N8R-immunized sera produced neutralizing activity against DENV2, and reduced enhancement of mortality as compared to WT-immunized sera. Thus, substitution of enhancing epitope residues can increase the immune response against viral infection while reducing the potential for ADE.

The antibodies induced by E protein of DENV play important roles in neutralizing effects and regulation of viral infection [21,38–40]. There are three structural domains (domain I, II, and III) in E protein. In previous reports, some mouse mAbs that bind to domain III of E protein were found to exhibit neutralizing activity and obstruct viral infection [21–23]. However, the anti-E or prM antibodies are cross-reactive and weakly neutralizing, which may enhance viral infection through ADE [26,36]. Here, we demonstrated that cross-reactive DB21-6 and DB39-2 against EDI-II have poor neutralizing activities against DENV1-4 (S2 Fig). In addition, we found that DB21-6 and DB39-2 have strong ADE activities *in vitro* (Fig 1). Previous studies have shown that anti-fusion loop 4G2 enhances viral infections in both *in vitro* ADE assays and AG129 mice [41]. We also observed that 4G2 has partially neutralizing activity against DENV1-4 (S2 Fig) and enhances *in vitro* viral infections at low antibody concentrations (Fig 1A). Notably, we also found that DB21-6 and DB39-2 enhanced DENV1-4 infection in K562 cells at high concentrations (Fig 1A). Furthermore, infection of DENV2 was enhanced to a greater extent by DB21-6 and DB39-2 than by 4G2 in THP-1 cells (Fig 1B). In addition, DB21-6 and DB39-2 enhanced mortality in AG129 mice (Fig 2A and 2B) and increases the viral loads in infected mice sera (Fig 2C). These results indicate that DB21-6 and DB39-2 have strong enhancing activity both *in vitro* and *in vivo*.

ADE is regarded as an important mechanism leading to the development of severe dengue disease, including DHF/DSS [5]. Cross-reactive and non-neutralizing antibodies binding to viruses can enhance infection of FcγR-bearing cells by ADE, resulting in increased viral load and/or production of cytokines [6]. High viral load is correlated with dengue disease severity and DHF [42,43]. Thus, there is a need to be able to confirm the presence of enhancing antibodies in dengue patient sera. Our results indicate that the competition percentages of DB21-6 and DB39-2 were significantly higher in DHF patient sera than those in DF patient sera (Fig 2D), suggesting that the higher levels of enhancing antibodies, DB21-6 and DB39-2, in serum samples of dengue patients are associated with severe dengue disease. We hypothesize that the DENV infected patients might suffer more severe symptoms, such as DHF, when the expression level of the enhancing antibodies is higher.

Identification of binding domain and epitope residues in the E protein may provide helpful information for investigation of neutralizing and enhancing mechanisms of dengue infection. Phage display is a powerful method for developing epitope-based diagnostics and identifying B-cell epitopes [21,44]. After screening a phage-displayed peptide library, we found that the phage clones selected using DB21-6 and DB39-2 mAbs displayed peptide sequences containing a consensus motif, N-R-x-x-V-E (Table 1). These displayed peptide sequences may be suitable for detecting enhancing antibodies in serum samples from dengue patients, and for providing information on the pathogenesis of dengue. By alignment of displayed peptide sequences and structural modeling, the candidate epitopes were predicted and verified using VLP mutants (Figs 3 and 4). The epitope residues of enhancing mAb DB21-6 are N8, R9, V12, and E13 in domain I of DENV2 E protein (Fig 4A and 4B), and the epitope residues of enhancing mAb DB39-2 are N8, R9, and E13 in domain I of DENV2 E protein (Fig 4A and 4B). We aligned the N8, R9, V12, and E13 residues, and found that these residues were conserved in DENV1-4 (S2 Table). Thus, cross-reactive DB21-6 and DB39-2 can bind to DENV1-4. A previous report indicated that G106 and L107 are the epitope residues of enhancing mAb 4G2 [16]. In our studies, we also confirmed that W101, G106, L107, and F108 in the fusion loop are the epitope residues of 4G2 (Fig 4A). The epitope residues recognized by 4G2 are different from those recognized by DB21-6 and DB39-2. These findings suggest that DB21-6/DB39-2 and 4G2 enhance DENV infection through different mechanisms. In addition, the enhancing epitopes of DB21-6 (N8, R9, V12, and E13) and DB39-2 (N8, R9, and E13) are novel and have not previously been reported. Therefore, further verification of these enhancing epitopes and the detailed molecular mechanism(s) by which these enhancing antibodies propagate dengue infection are worth investigating through cryo-electron microscopy (cryo-EM).

The E protein is targeted by most reported dengue vaccines, and is thus regarded as an important target [28]. Sanofi Pasteur published data from a phase III study on tetravalent dengue vaccine, which conferred moderate protection (56%) against dengue disease [45]. Furthermore, the vaccine provided low protection (35%) against DENV2, but more than 75% protection against DENV3 and 4, and 50% against DENV1. Improvements in vaccine efficacy and the effect of the substitution of the enhancing epitope on safety are yet to be examined. Previous studies have shown that DNA vaccine candidates against DENV1 or DENV2 with substitutions in the fusion loop (at G106 and L107) and the cross-reactive epitopes of EDIII (at K310, E311, and P364) confer protective immunity [46,47]. In addition, enhancement of mortality by enhancing antibodies against the fusion loop was reduced in mice immunized with such vaccines. In this study, we have identified new enhancing antibodies and a novel enhancing epitope that are different from those previously reported. Mutations at R9, V12, and E13 may change the structure of E protein and affect VLP secretion. However, our VLP-capture ELISA results suggest that the N8R substitution does not affect DENV2 VLP secretion (Fig 4D), which is crucial for its use in immunization. Moreover, we used an *in vitro* neutralizing assay and *in vivo* protection assays to show that both WT- and N8R-immunized sera exerted protective activities against DENV2 (Fig 5). Interestingly, N8R-immunized sera had higher *in vitro* neutralizing activity and *in vivo* protective activity than the WT-immunized sera (Fig 5B–5E). These results suggest that immunization with the N8R DNA vaccine may increase neutralizing and protective immunity against DENV2.

An earlier investigation used mouse-adapted DENV2 S221 to study severe dengue disease via ADE in AG129 mice [30]. Here, we passively transferred diluted vector-, WT-, or N8R-immunized sera, and then challenged AG129 mice with DENV2 S221. WT- and N8R-immunized sera were protective at a 1:25 dilution, as compared to vector-immunized sera. However, the mortality of mice was enhanced by treatment with WT-immunized sera at a 1:100 dilution, as compared to treatment with vector-immunized sera. Importantly, the mortality of mice treated with N8R-immunized sera at a 1:100 dilution was not enhanced (Fig 6B). When the dilution was increased to 1:400, no enhanced mortality was observed (Fig 6C). Our results indicate that substituting the enhancing epitope can reduce the ADE phenomenon and increase protective activity *in vivo*.

In this study, substitution of enhancing epitope and preservation of neutralizing epitope in immunized mice provide protective immunity. Such an approach would redirect immunodominance (Fig 6E) and improve immunogenicity by satisfying the required neutralizing occupancy [48]. In summary, we have identified a novel enhancing epitope, enabling us to reduce the potential for ADE through N8R substitution in DENV2 E protein. This may be a viable approach for developing new dengue vaccines that can increase the anti-DENV immune response.

## Supporting Information

S1 Fig Characterization of DB21-6 and DB39-2 against DENV and E protein of DENV2 by immunofluorescence assay (IFA). (A) BHK-21 cells were infected with DENV1 (MOI = 1), DENV2 (MOI = 1), DENV3 (MOI = 10), or DENV4 (MOI = 1). After 2 days, the infected cells were detected using DB21-6 and DB39-2. Uninfected cells (Mock) and NMIgG were used as negative controls. The results are shown at 400× magnification. (B and C) DENV2 E, comprising amino acids 1–400 of the E protein, was cloned into the pcDNA3.1 plasmid. DENV2 EDI-II (amino acids 1–295) and EDIII (amino acids 295–400) were also inserted into the pcDNA3.1 plasmid. After transfection, IFA was used to reveal that DB21-6 and DB39-2 recognized BHK-21 cells expressing DENV2 E (B) or EDI-II (C) protein.
(TIF)

S2 Fig Evaluation of neutralizing activity of mAbs against DENV1-4. (A) The neutralizing activity of DB21-6, DB39-2, or 4G2 against DENV1-4 was examined with inhibition assays. DENV1 (MOI = 5), DENV2 (MOI = 1), DENV3 (MOI = 5), or DENV4 (MOI = 1) was incubated with mAbs at 4°C for 1 hour, and then used to infect BHK-21 cells. After 3 days, the cells were fixed and stained with 4G2. Titers are expressed as inhibition percentages. Data shown are from one representative experiment of two independent experiments. (B) Summary of 50% inhibition concentrations of DB21-6, DB39-2, and 4G2 against DENV1-4. The concentrations resulting in 50% inhibition were analyzed using GraphPad Prism 5.
(TIF)

S3 Fig *In vitro* measurement of mAb-mediated ADE of DENV2 S221 in K562 and THP-1 cells. (A) DENV2 S221 (MOI = 5) was incubated with dilutions of mAbs for 1 hour at 4°C, and the resulting mixture was used to infect K562 cells. After 3 days, the cells were stained with 4G2, and analyzed by flow cytometry. (B) DENV2 S221 (MOI = 10) was incubated with dilutions of mAbs for 1 hour at 4°C, and the resulting mixture was used to infect THP-1 cells. After 3 days, the cells were stained with 4G2, and analyzed by flow cytometry.
(TIF)

S4 Fig Competition assay using mAbs and patient sera. Competition for binding to DENV2 between mAbs and patient sera at a dilution of 1:50 (A) or 1:200 (B). The percentage of competition is shown. Unpaired Student’s *t* tests were used to calculate *P* values (*P<0.05, **P<0.01, ***P<0.001, NS not significant).
(TIF)

S5 Fig Evaluation of humoral immune responses against DENV2 by ELISA. (A) Mice were immunized with vector control, WT, or N8R plasmids at three-week intervals. The serum samples were collected after one, two, and three immunizations, and pooled. Next, the sera were evaluated using plates containing C6/36 cells infected with DENV2 16681. (B) The collected sera were diluted 1:200 and detected with anti-IgG1 or IgG2a antibodies. The OD was measured at 490 nm. The data are presented as mean values. (C) IgG1/IgG2a ratios. (D) After the 3^rd^ immunization, immunized sera were collected and examined by ELISA at weeks 6, 9, 12, and 15. The OD was measured at 490 nm. The data are presented as mean values. The *P* values (***P<0.001) were analyzed using two-way ANOVA with Bonferroni *post-hoc* test.
(TIF)

S6 Fig Neutralization of infections with different DENV2 strains. (A) BHK-21 cells were infected with DENV2 16681, PL046, Malaysia 07587, or NGC at an MOI of 1, 1, 1, or 10, respectively, at 37°C for 2 hours. After 3 days, the cells were fixed, stained with 4G2, and RPE-conjugated goat anti-mouse IgG. The percentages of infected cells were analyzed by flow cytometry. BHK-21 cells were used as a negative control (Mock). PE, phycoerythrin. FSC, forward scatter. (B) Serial dilutions of 3^rd^ immunized pooled sera were incubated with DENV2 16681, PL046, Malaysia 07587, or NGC at an MOI of 1, 1, 1, or 10, respectively, at 4°C for 1 hour. The resulting mixtures were then used to infect BHK-21 cells. After 3 days, the cells were stained with 4G2, and analyzed by flow cytometry.
(TIF)

S1 Table The DENV2-infected patient serum samples used in this study. (DOCX)

S2 Table Comparison of the amino acid sequences of the EDI-II proteins of DENV1, 2, 3, and 4. (DOCX)

S3 Table The database, gene / protein and accession / ID numbers were mentioned in the text. (DOCX)
