# Supplementary figures and images for: An Epitope-Substituted DNA Vaccine Improves Safety and Immunogenicity against Dengue Virus Type 2
Source: PLoS Negl Trop Dis. 2015 Jul 2;9(7):e0003903. doi: 10.1371/journal.pntd.0003903 (PMC4489899; doi:10.1371/journal.pntd.0003903)

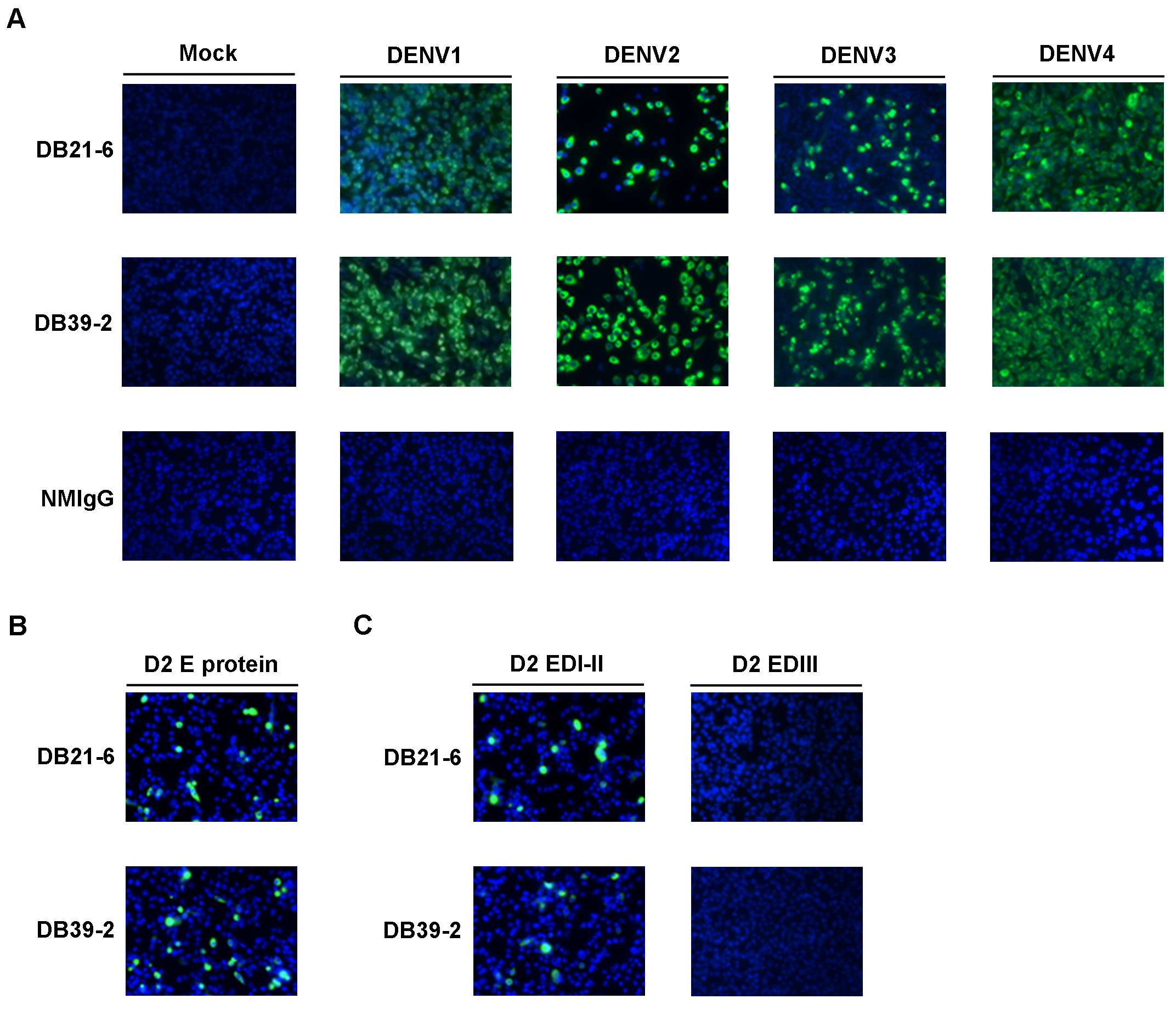

Supplement: S1 Fig — (A) BHK-21 cells were infected with DENV1 (MOI = 1), DENV2 (MOI = 1), DENV3 (MOI = 10), or DENV4 (MOI = 1). After 2 days, the infected cells were detected using DB21-6 and DB39-2. Uninfected cells (Mock) and NMIgG were used as negative controls. The results are shown at 400× magnification. (B and C) DENV2 E, comprising amino acids 1–400 of the E protein, was cloned into the pcDNA3.1 plasmid. DENV2 EDI-II (amino acids 1–295) and EDIII (amino acids 295–400) were also inserted into the pcDNA3.1 plasmid. After transfection, IFA was used to reveal that DB21-6 and DB39-2 recognized BHK-21 cells expressing DENV2 E (B) or EDI-II (C) protein. (TIF) [file pntd.0003903.s001.tif]

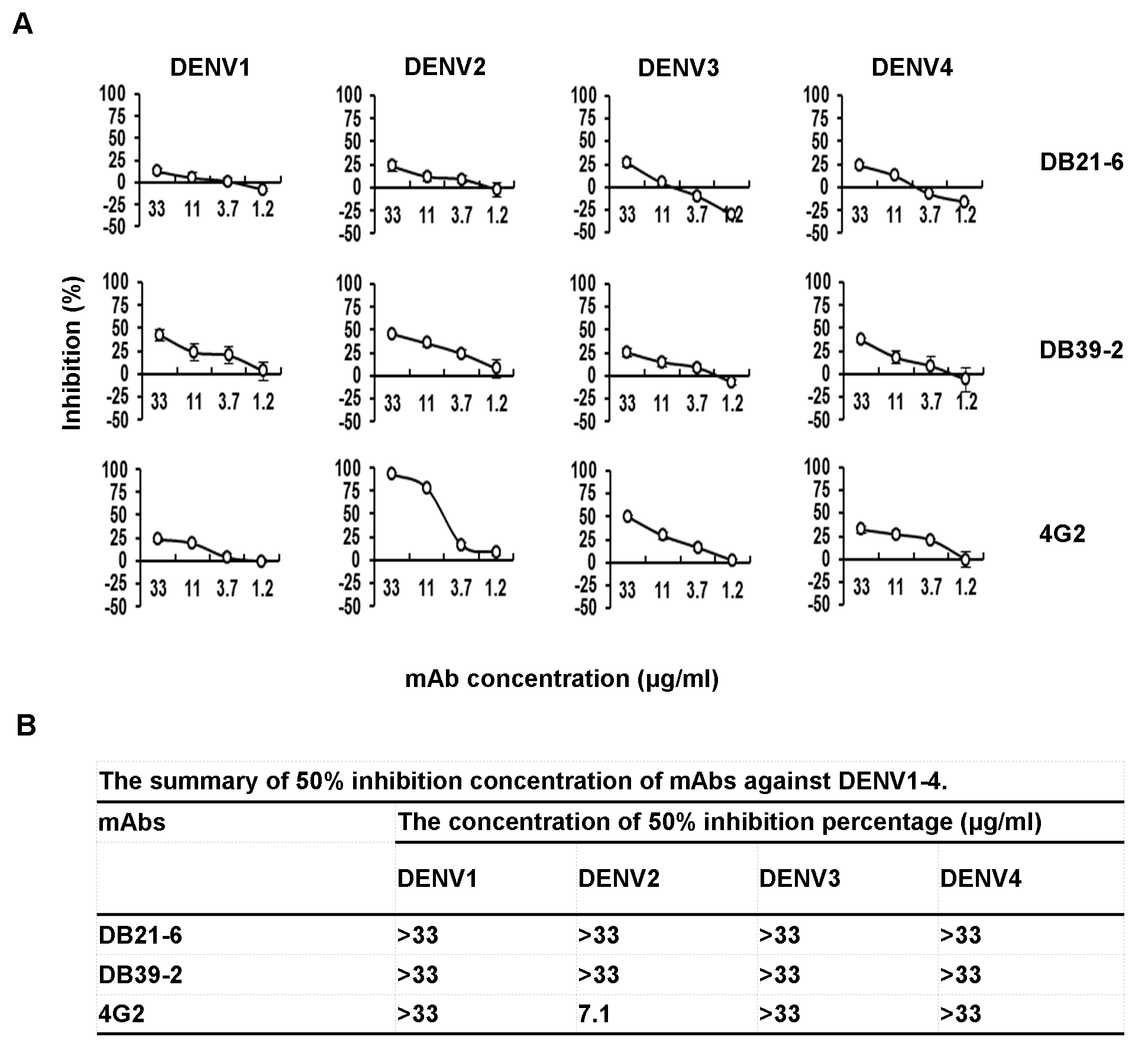

Supplement: S2 Fig — (A) The neutralizing activity of DB21-6, DB39-2, or 4G2 against DENV1-4 was examined with inhibition assays. DENV1 (MOI = 5), DENV2 (MOI = 1), DENV3 (MOI = 5), or DENV4 (MOI = 1) was incubated with mAbs at 4°C for 1 hour, and then used to infect BHK-21 cells. After 3 days, the cells were fixed and stained with 4G2. Titers are expressed as inhibition percentages. Data shown are from one representative experiment of two independent experiments. (B) Summary of 50% inhibition concentrations of DB21-6, DB39-2, and 4G2 against DENV1-4. The concentrations resulting in 50% inhibition were analyzed using GraphPad Prism 5. (TIF) [file pntd.0003903.s002.tif]

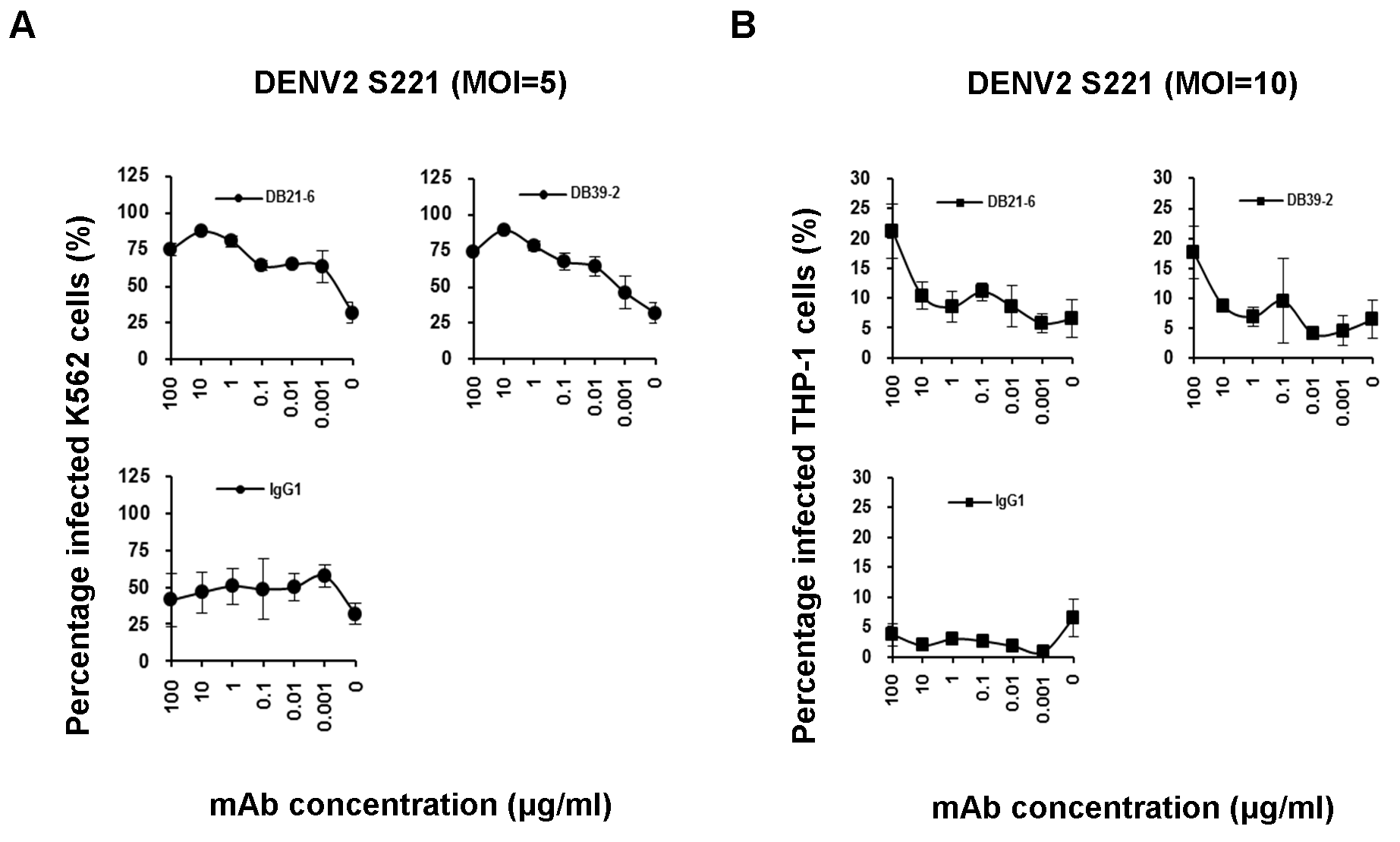

Supplement: S3 Fig — (A) DENV2 S221 (MOI = 5) was incubated with dilutions of mAbs for 1 hour at 4°C, and the resulting mixture was used to infect K562 cells. After 3 days, the cells were stained with 4G2, and analyzed by flow cytometry. (B) DENV2 S221 (MOI = 10) was incubated with dilutions of mAbs for 1 hour at 4°C, and the resulting mixture was used to infect THP-1 cells. After 3 days, the cells were stained with 4G2, and analyzed by flow cytometry. (TIF) [file pntd.0003903.s003.tif]

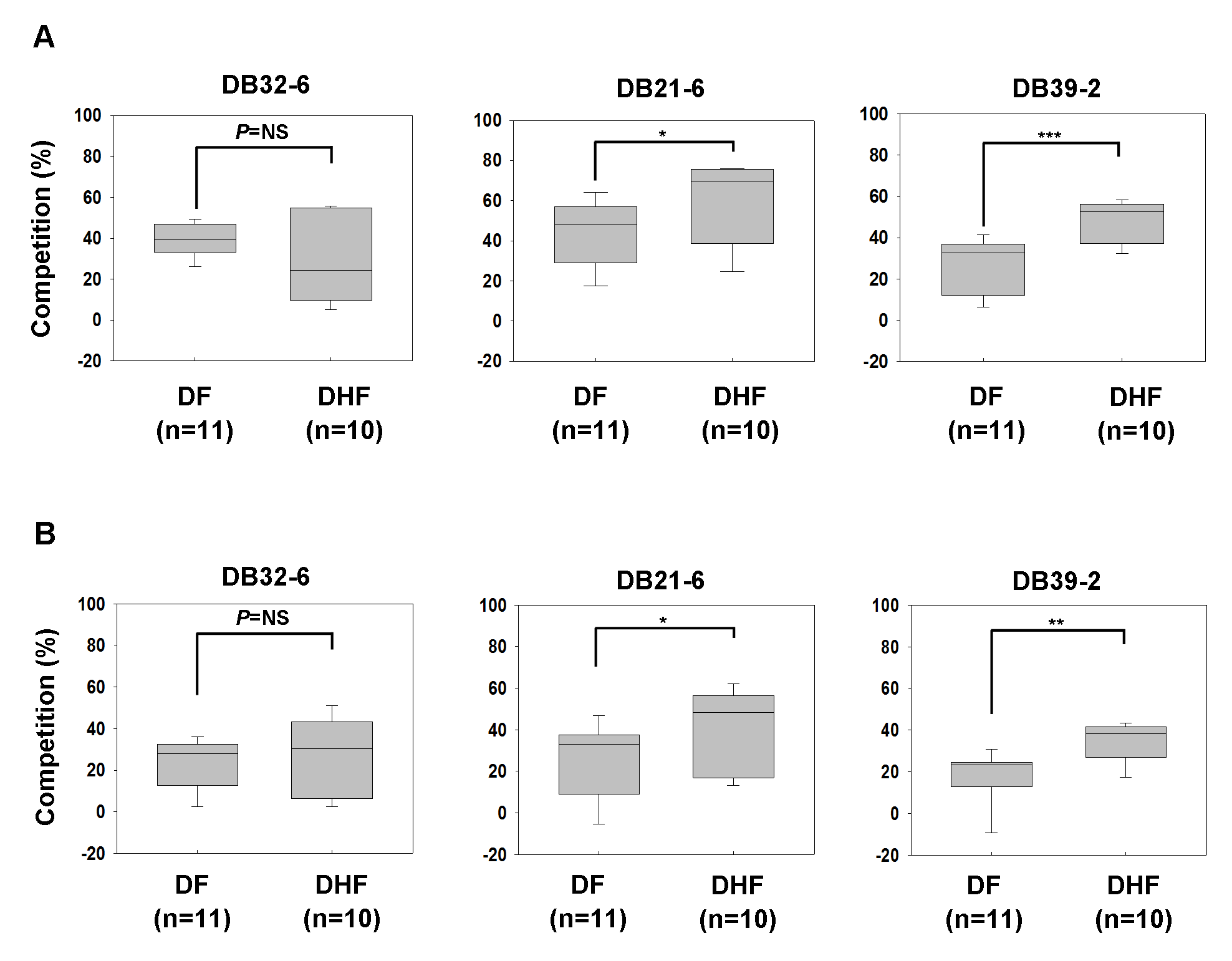

Supplement: S4 Fig — Competition for binding to DENV2 between mAbs and patient sera at a dilution of 1:50 (A) or 1:200 (B). The percentage of competition is shown. Unpaired Student’s t tests were used to calculate P values (*P<0.05, **P<0.01, ***P<0.001, NS not significant). (TIF) [file pntd.0003903.s004.tif]

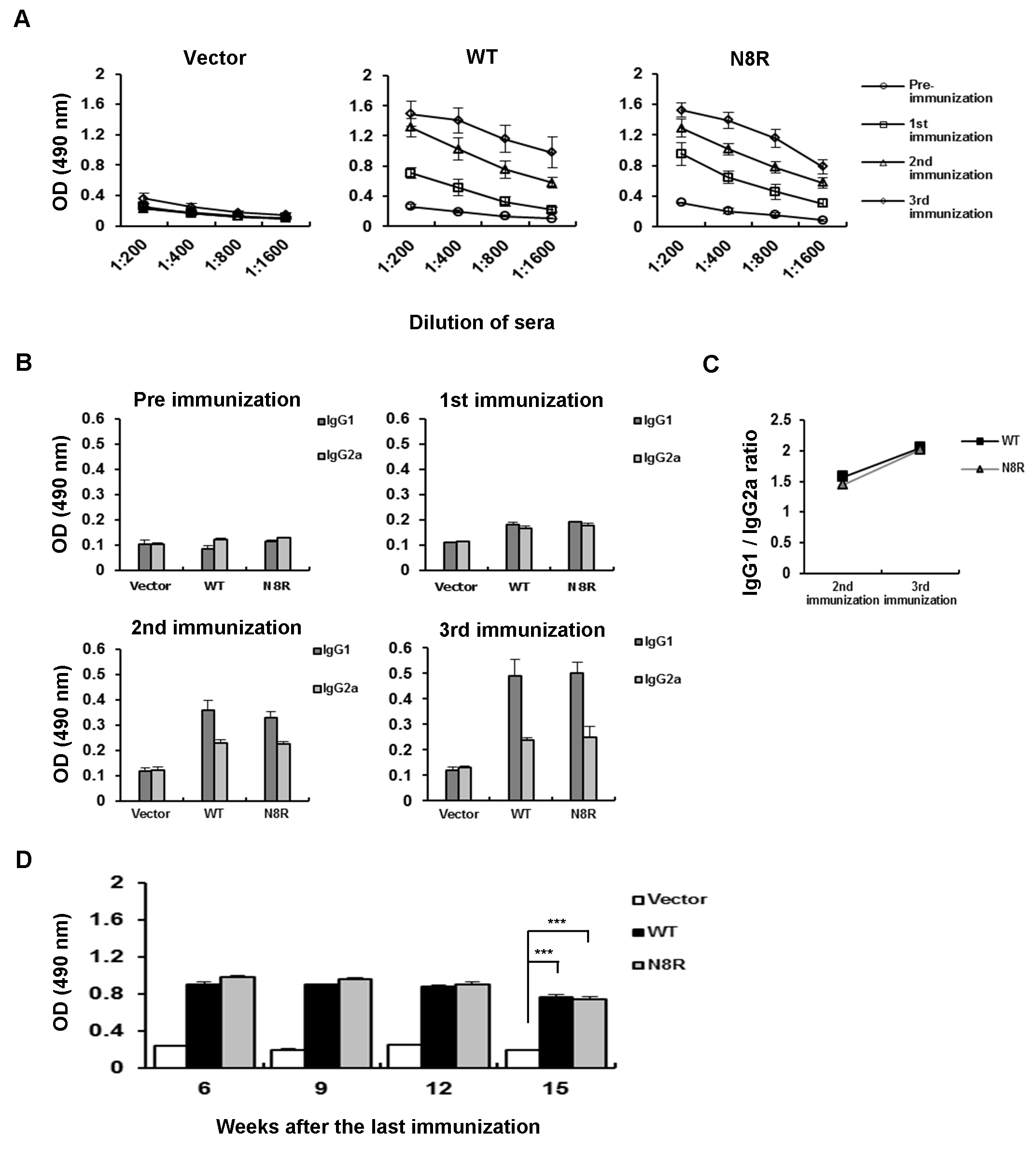

Supplement: S5 Fig — (A) Mice were immunized with vector control, WT, or N8R plasmids at three-week intervals. The serum samples were collected after one, two, and three immunizations, and pooled. Next, the sera were evaluated using plates containing C6/36 cells infected with DENV2 16681. (B) The collected sera were diluted 1:200 and detected with anti-IgG1 or IgG2a antibodies. The OD was measured at 490 nm. The data are presented as mean values. (C) IgG1/IgG2a ratios. (D) After the 3rd immunization, immunized sera were collected and examined by ELISA at weeks 6, 9, 12, and 15. The OD was measured at 490 nm. The data are presented as mean values. The P values (***P<0.001) were analyzed using two-way ANOVA with Bonferroni post-hoc test. (TIF) [file pntd.0003903.s005.tif]

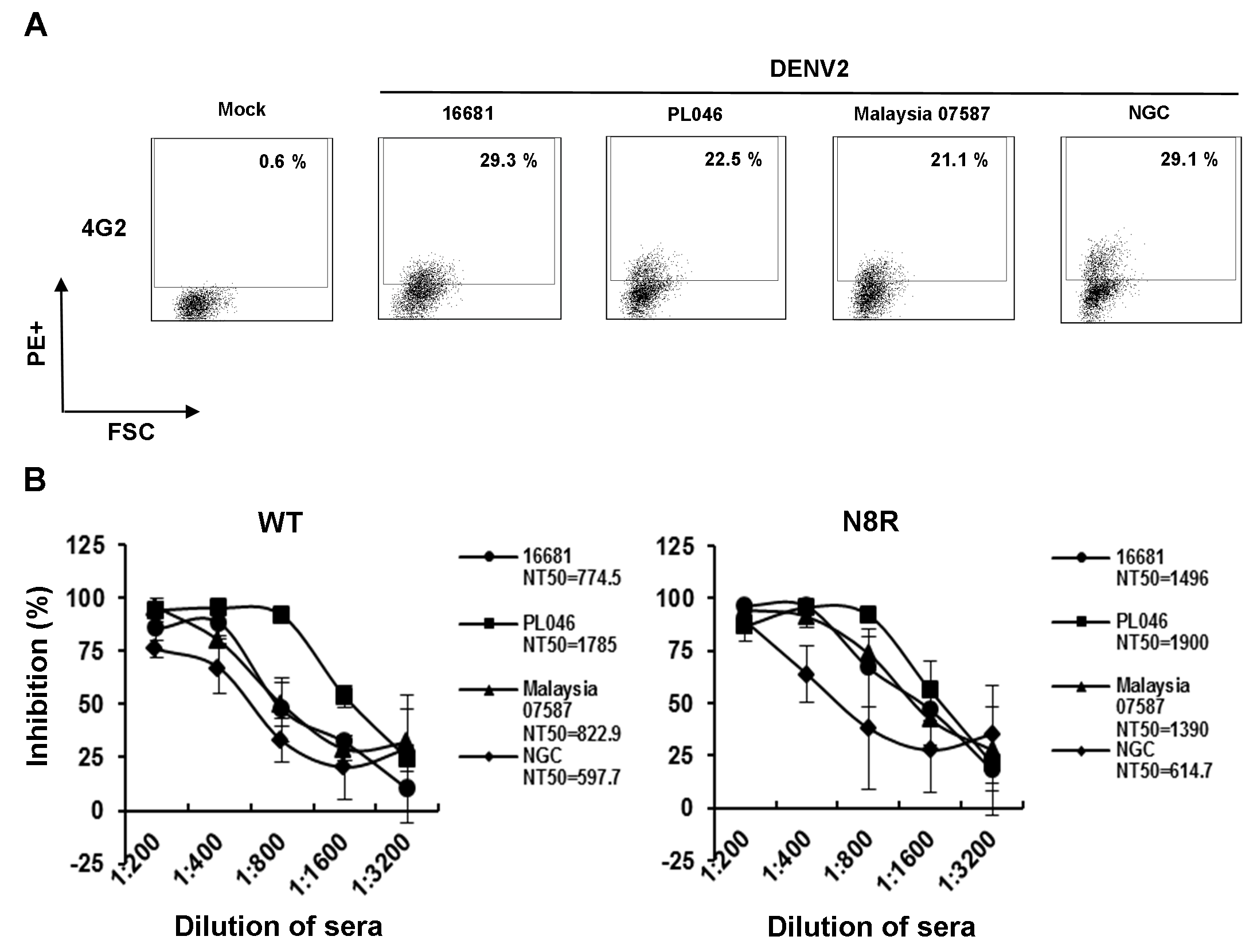

Supplement: S6 Fig — (A) BHK-21 cells were infected with DENV2 16681, PL046, Malaysia 07587, or NGC at an MOI of 1, 1, 1, or 10, respectively, at 37°C for 2 hours. After 3 days, the cells were fixed, stained with 4G2, and RPE-conjugated goat anti-mouse IgG. The percentages of infected cells were analyzed by flow cytometry. BHK-21 cells were used as a negative control (Mock). PE, phycoerythrin. FSC, forward scatter. (B) Serial dilutions of 3rd immunized pooled sera were incubated with DENV2 16681, PL046, Malaysia 07587, or NGC at an MOI of 1, 1, 1, or 10, respectively, at 4°C for 1 hour. The resulting mixtures were then used to infect BHK-21 cells. After 3 days, the cells were stained with 4G2, and analyzed by flow cytometry. (TIF) [file pntd.0003903.s006.tif]
