# Supplementary material for: An Epitope-Substituted DNA Vaccine Improves Safety and Immunogenicity against Dengue Virus Type 2
Source: PLoS Negl Trop Dis. 2015 Jul 2;9(7):e0003903. doi: 10.1371/journal.pntd.0003903 (PMC4489899; doi:10.1371/journal.pntd.0003903)
Supplement: S1 Table — (DOCX) [file pntd.0003903.s007.docx]

| **S1 Table. The DENV2-infected patient serum samples used in this study.** | | | |
| --- | --- | --- | --- |
| Patient number | Severity^a^ | Days post onset of symptoms | Primary /Secondary infection |
| 1 | DHF | 17 | Primary |
| 2 | DHF | 22 | Primary |
| 3 | DHF | 5 | Secondary |
| 4 | DHF | 6 | Secondary |
| 5 | DHF | 7 | Secondary |
| 6 | DHF | 8 | Secondary |
| 7 | DHF | 4 | Secondary |
| 8 | DHF | 7 | Secondary |
| 9 | DHF | 8 | Secondary |
| 10 | DHF | 4 | Secondary |
| 11 | DF | 6 | Primary |
| 12 | DF | 10 | Primary |
| 13 | DF | 11 | Primary |
| 14 | DF | 7 | Primary |
| 15 | DF | 6 | Secondary |
| 16 | DF | 6 | Secondary |
| 17 | DF | 5 | Secondary |
| 18 | DF | 7 | Secondary |
| 19 | DF | 7 | Secondary |
| 20 | DF | 7 | Secondary |
| 21 | DF | 4 | Secondary |
| ^a^DHF, dengue hemorrhagic fever; DF, dengue fever. | | | |
