# Supplementary material for: An Epitope-Substituted DNA Vaccine Improves Safety and Immunogenicity against Dengue Virus Type 2
Source: PLoS Negl Trop Dis. 2015 Jul 2;9(7):e0003903. doi: 10.1371/journal.pntd.0003903 (PMC4489899; doi:10.1371/journal.pntd.0003903)
Supplement: S2 Table — (DOCX) [file pntd.0003903.s008.docx]

| **S2 Table. Comparison of the amino acid sequences of the EDI-II proteins of DENV1, 2, 3, and 4^a^** | | | | | |
| --- | --- | --- | --- | --- | --- |
| Serotypes of DENV (strain) | Accession number | Amino acid of EDI-II protein | | | |
|  | (GenBank) | 8^b, c^ | 9^b, c^ | 12^b^ | 13^b, c^ |
| DENV2 (16681) | AAB58782 | **N** | **R** | **V** | **E** |
| DENV2 (NGC) | AAA42941 | **N** | **R** | **V** | **E** |
| DENV2 (PL046) | AHZ61501 | **N** | **R** | **V** | **E** |
| DENV2 (PM33974) | ABO33322 | **N** | **R** | **V** | **E** |
| DENV2 (IQT2913) | AAD32963 | **N** | **R** | **V** | **E** |
| DENV2 (ThD2_0038_74) | ABA61185 | **N** | **R** | **V** | **E** |
| DENV2 (ThD2_0168_79) | ABA61184 | **N** | **R** | **V** | **E** |
| DENV2 (ThD2_0498_84) | ABA61183 | **N** | **R** | **V** | **E** |
| DENV2 (ThD2_0263_95) | ABA61179 | **N** | **R** | **V** | **E** |
| DENV2 (ThD2_0017_98) | ABA61178 | **N** | **R** | **V** | **E** |
| DENV2 (ThD2_0284_90) | ABA61180 | **N** | **R** | **V** | **E** |
| DENV2 (Jamaica/N.1409) | AAA42942 | **N** | **R** | **V** | **E** |
| DENV2 (TSV01) | AAK67712 | **N** | **R** | **V** | **E** |
| DENV2 (98900663 DHF DV-2) | BAD42415 | **N** | **R** | **V** | **E** |
| DENV2 (Tonga/74) | AAV70829 | **N** | **R** | **V** | **E** |
| DENV2 (I348600) | AAW31413 | **N** | **R** | **V** | **E** |
| DENV1 (Hawaii) | AIU47321 | **N** | **R** | **V** | **E** |
| DENV1 (16007) | AAF59977 | **N** | **R** | **V** | **E** |
| DENV3 (ThD3_0183_85) | AAV88402 | **N** | **R** | **V** | **E** |
| DENV3 (H87) | AAA99437 | **N** | **R** | **V** | **E** |
| DENV4 (H241) | AAX48017 | **N** | **R** | **V** | **E** |
| DENV4 (B5) | AAG30148 | **N** | **R** | **V** | **E** |
| DENV4 (ThD4_0348_91) | AAU89377 | **N** | **R** | **V** | **E** |
| DENV4 (ThD4_0087_77) | AAU89378 | **N** | **R** | **V** | **E** |
| DENV4 (ThD4_0485_01) | AAU89379 | **N** | **R** | **V** | **E** |
| DENV4 (ThD4_0734_00) | AAU89380 | **N** | **R** | **V** | **E** |
| DENV4 (814669) | AAK01233 | **N** | **R** | **V** | **E** |
| DENV4 (Indonesia 1976) | AAB70680 | **N** | **R** | **V** | **E** |
| DENV4 (ThD4_0476_97) | AAU89375 | **N** | **R** | **V** | **E** |
| ^a^EDI-II protein of strains of the four dengue virus serotypes are aligned. Single letter amino acid abbreviations are shown. | | | | | |
| ^b^The critical residues are shown in boldface type, and were identified by loss of binding for DB21-6. | | | | | |
| ^c^The critical residues are shown in boldface type, and were identified by loss of binding for DB39-2. | | | | | |
