# Supplementary material for: An Epitope-Substituted DNA Vaccine Improves Safety and Immunogenicity against Dengue Virus Type 2
Source: PLoS Negl Trop Dis. 2015 Jul 2;9(7):e0003903. doi: 10.1371/journal.pntd.0003903 (PMC4489899; doi:10.1371/journal.pntd.0003903)
Supplement: S3 Table — (DOCX) [file pntd.0003903.s009.docx]

| **S3 Table. The database, gene / protein and accession / ID numbers were mentioned in the text.** | | |
| --- | --- | --- |
| Gene / Protein | Database | Accession / ID number |
| DENV2 E protein | Protein Data Bank | 1OAN |
| DENV2 (16681) | GenBank | AAB58782 |
| DENV2 (NGC) | GenBank | AAA42941 |
| DENV2 (PL046) | GenBank | AHZ61501 |
| DENV2 (PM33974) | GenBank | ABO33322 |
| DENV2 (IQT2913) | GenBank | AAD32963 |
| DENV2 (ThD2_0038_74) | GenBank | ABA61185 |
| DENV2 (ThD2_0168_79) | GenBank | ABA61184 |
| DENV2 (ThD2_0498_84) | GenBank | ABA61183 |
| DENV2 (ThD2_0263_95) | GenBank | ABA61179 |
| DENV2 (ThD2_0017_98) | GenBank | ABA61178 |
| DENV2 (ThD2_0284_90) | GenBank | ABA61180 |
| DENV2 (Jamaica/N.1409) | GenBank | AAA42942 |
| DENV2 (TSV01) | GenBank | AAK67712 |
| DENV2 (98900663 DHF DV-2) | GenBank | BAD42415 |
| DENV2 (Tonga/74) | GenBank | AAV70829 |
| DENV2 (I348600) | GenBank | AAW31413 |
| DENV1 (Hawaii) | GenBank | AIU47321 |
| DENV1 (16007) | GenBank | AAF59977 |
| DENV3 (ThD3_0183_85) | GenBank | AAV88402 |
| DENV3 (H87) | GenBank | AAA99437 |
| DENV4 (H241) | GenBank | AAX48017 |
| DENV4 (B5) | GenBank | AAG30148 |
| DENV4 (ThD4_0348_91) | GenBank | AAU89377 |
| DENV4 (ThD4_0087_77) | GenBank | AAU89378 |
| DENV4 (ThD4_0485_01) | GenBank | AAU89379 |
| DENV4 (ThD4_0734_00) | GenBank | AAU89380 |
| DENV4 (814669) | GenBank | AAK01233 |
| DENV4 (Indonesia 1976) | GenBank | AAB70680 |
| DENV4 (ThD4_0476_97) | GenBank | AAU89375 |
